# Conservation planning for species recovery under the Endangered Species Act: A case study with the Northern Spotted Owl

**DOI:** 10.1371/journal.pone.0210643

**Published:** 2019-01-14

**Authors:** Jeffrey R. Dunk, Brian Woodbridge, Nathan Schumaker, Elizabeth M. Glenn, Brendan White, David W. LaPlante, Robert G. Anthony, Raymond J. Davis, Karl Halupka, Paul Henson, Bruce G. Marcot, Michele Merola-Zwartjes, Barry R. Noon, Martin G. Raphael, Jody Caicco, Dan L. Hansen, Mary Jo Mazurek, James Thrailkill

**Affiliations:** 1 Department of Environmental Science and Management, Humboldt State University, 1 Harpst St. Arcata, CA, United States of America; 2 US Fish and Wildlife Service, Western Golden Eagle Conservation, U.S Fish and Wildlife Service, Corvallis, OR, United States of America; 3 US Environmental Protection Agency, Environmental Research Lab, Corvallis, OR, United States of America; 4 US Fish and Wildlife Service, Oregon Fish and Wildlife Office, Portland, OR, United States of America; 5 Natural Resources Geospatial, sMontague, CA, United States of America; 6 Department of Fisheries and Wildlife, Oregon State University, Corvallis, OR, United States of America; 7 US Forest Service, Forestry Sciences Laboratory, Corvallis, OR, United States of America; 8 US Fish and Wildlife Service, Wenatchee, WA, United States of America; 9 USDA Forest Service, Pacific Northwest Research Station, Portland, OR, United States of America; 10 US Fish and Wildlife Service, Region 1 Endangered Species Listing Division, Portland, OR, United States of America; 11 Department of Fish, Wildlife, and Conservation Biology and Graduate Degree Program in Ecology, Colorado State University, Fort Collins, CO, United States of America; 12 USDA Forest Service, Pacific Northwest Research Station, Olympia, WA, United States of America; 13 Humboldt State University Sponsored Programs Foundation, Arcata, CA, United States of America; 14 USDI Fish and Wildlife Service, Roseburg Field Office, Roseburg, OR, United States of America; California State University Sacramento, UNITED STATES

## Abstract

The northern spotted owl (*Strix occidentalis caurina*) was listed as threatened under the U.S. Endangered Species Act (ESA) in 1990. We applied modern spatial conservation theory and models to evaluate several candidate critical habitat networks, and sought an efficient conservation solution that encompassed the highest value lands for spotted owl recovery rather than maximizing the total area of potential critical habitat. We created a map of relative habitat suitability, which served as input to the spatial conservation prioritization program Zonation. We used the spatially-explicit individual-based population model HexSim to estimate and compare simulated spotted owl population outcomes among a suite of candidate critical habitat networks that varied in size and spatial arrangement under alternative scenarios of future habitat suitability and barred owl (*S*. *varia*) effects. We evaluated simulated spotted owl population outcomes, including total population size, and extinction and quasi-extinction likelihoods for 108 combinations of candidate critical habitat networks by habitat change by barred owl scenarios, both range-wide and within 11 distinct portions of the owl’s range. Barred owl encounter rates and the amount and suitability of habitat had substantial effects on simulated spotted owl populations. When barred owl encounter rates were high, changes in the amount and suitability of habitat had minimal impacts on population performance. Under lowered barred owl encounter rates, candidate critical habitat networks that included most existing high suitability habitat supported a high likelihood of long-term population persistence. Barred owls are currently the primary driving force behind poor population performance of NSOs; however, our models demonstrated that a sufficient area of high suitability habitat remains essential for recovery when effects of barred owls can be reduced. The modeling approach we employed is sufficiently flexible to incorporate new information about spotted owls as it becomes available and could likely be applied to conservation planning for other species.

## Introduction

Methods for spatially explicit conservation planning have increased rapidly over the last several decades. For example, the seminal paper “Systematic Conservation Planning” [1] had been cited 4,819times (Google Scholar accessed 19 Aug 2018). It was estimated that more than 5,000 papers had been published on various aspects of conservation planning since the mid-1980s [2]. The quantity and quality of datasets on species’ locations, broad-scale patterns of land-cover, and fine-scale environmental data have increased dramatically over this period. Coupled with increases in computing power, complex spatial analyses linking species location data to environmental factors are now possible. Given this capability along with access to free software for conducting analyses, it is not surprising that conservation plans are generated at a rate that greatly exceeds their implementation [3]. In cases where conservation planning is conducted under the auspices of government regulations, successful implementation is also reliant on the degree to which the conservation planning approach and modeling tools address statutory requirements (e.g., the Endangered Species Act).

To date, modern conservation planning methods have rarely been used for identifying and evaluating critical habitat for organisms listed as threatened or endangered under the United States’ Endangered Species Act. Critical habitat represents the areas within the geographic area occupied by a species listed under the Endangered Species Act (ESA) that contain the physical and biological features that are essential to conservation of the species and that may need special management or protection (Endangered Species Act, Section 1532). The goal of our modeling effort was to identify and evaluate the potential effectiveness of candidate critical habitat networks (hereafter, networks) for the northern spotted owl (*Strix occidentalis caurina*; hereafter, spotted owl), which was listed in 1990 as threatened under the ESA. Our work was a response to statutory requirements of the ESA which include the development of a recovery plan and the designation of critical habitat. We begin our paper by developing and articulating the theoretical framework used to identify networks by 1) linking the concepts of habitat and niche; 2) linking systematic conservation planning, as a process, to identifying spatially explicit networks; and 3) formalizing a science-based approach to address the legal and policy-mandated requirements of critical habitat designation.

Because critical habitat as defined in the ESA is not explicitly linked to a species’ demography, we clarify how we viewed critical habitat in our analyses. Grinnell [4] and Hutchinson [5] viewed niches as a property of a species, so they are necessarily viewed as a species-specific concept [6]. In light of a combined Grinnell-Hutchinson view of the niche, we define habitat as areas that possess features of the environment that, on average, allow a species to experience a positive growth rate, and thus must allow for occupancy, survival, and reproduction [6]. In practice, the relationship between habitat and demography is seldom known, so this assumption generally remains untested (but see [7, 8, 9]). Estimates of the niche based solely on observations in nature pertain to the realized, rather than to the fundamental, niche of a species, accounting for the influence of other organisms such as predators and competitors. In addition, we assume that properties of habitat determine the abundance of the focal organism in any given area and that these properties can be measured (e.g., [10]).

Habitat is species-specific as well as a *spatial* and a *temporal concept*. By definition, no two species have exactly the same habitat requirements just as no two species’ niches are identical. Finally, habitat is a *multi-dimensional concept*—habitat is characterized by multiple environmental factors (e.g., climate, vegetation, prey, terrain ruggedness). Within a specific area, habitat is the collection of resources and environmental conditions needed to support survival and reproduction of the focal organism and support population persistence over time. Thus, habitat is a specific combination of both biotic and abiotic components and processes that allow continuing occupancy of the environment by an organism [6].

The systematic approach to conservation planning is a step-down process [1] that has been used in aquatic and terrestrial ecosystems for a wide diversity of species (e.g., [11–13]). Systematic conservation planning attempts to make the best use of existing information, recognizing uncertainties and limited funds. It is, by design, adaptable to new information, insights, or conditions [1].

To be most effective, conservation network planning would include: 1) a good understanding of habitat requirements for the species of interest, including how variation in habitat types, amounts, and juxtapositions influence population growth [14]; 2) a method for translating knowledge of habitat requirements into a spatially-explicit map representing variation in habitat suitability at a scale relevant to the species [15, 16]; 3) an understanding of each species’ adaptive demographic response to multiple networks under both current and future conditions; and 4) performance metrics that can be used to rank networks in terms of their relative risk to species persistence or likelihood of achieving desired conservation outcomes. Conservation network planning typically uses a variety of modeling tools to determine species' habitat and resource requirements [17, 18]. For evaluating potential critical habitat for the spotted owl, we chose this approach to provide decision makers with a set of scientifically defensible outputs from a series of plausible scenarios (viz., changes in habitat suitability and barred owl [*S*. *varia*] presence) among candidate networks from which to make decisions on spotted owl critical habitat designation.

According to the ESA, critical habitat is defined as “(1) The specific areas within the geographical area occupied by the species, at the time it is listed in accordance with the Act, on which are found those physical or biological features: (a) essential to the conservation of the species; and (b) which may require special management considerations or protection; and (2) specific areas outside the geographic area occupied by the species at the time it is listed, upon a determination that such areas are essential for the conservation of the species.”

We operationally defined critical habitat as the area on the landscape needed for the spotted owl to reach an appropriate population size and geographic distribution so that its risk of extinction meets specified recovery criteria (e.g., <5% chance of extinction over the next 100 years). Implicit in this definition is a set of scale-dependent biological criteria that must be met by critical habitat. These criteria include:

1. At the *individual organism scale*, the habitat provides the resources and physical conditions necessary for individual spotted owls to survive and reproduce.
2. At the *local population scale*, habitat must be sufficiently extensive and connected so that it has a high probability of supporting a local population of sufficient size to be resilient to natural and human disturbance events and not experience local extinction over the time frame of interest.
3. At the *geographic range scale*, habitat must be sufficiently extensive at the scale of the spotted owl's geographic range so that it is highly unlikely that all local populations will simultaneously experience extinction events. That is, the dynamics of local populations are asynchronous as a consequence of spatial redundancy of geographic range-scale critical habitat designations. This would constitute a viable metapopulation [19].

Given a recovery objective (e.g., <5% chance of extinction over the next 100 years), critical habitat must address all three spatial scales resulting in a habitat network that, on average, results in positive growth rates, while also being resilient to disturbances and/or changes such as wildfire or climate change.

The spotted owl is a conservation icon and one of the most studied of all imperiled species [20]. The spotted owl’s selection of forests characterized by old, large, economically valuable trees, coupled with its large area requirements (home ranges >1,000 ha in many areas; [21]), has resulted in controversy over how to conserve the species while preserving the economies of timber-dependent communities [22, 23]. The chronology of spotted owl conservation efforts is well-documented [24–26] and thus, we will only briefly elaborate on it herein.

Early research on the spotted owl demonstrated a strong association of nesting and roosting sites with areas of late seral forests [27–30], which was corroborated by subsequent studies across the range of the species [21]. A notable exception to the general pattern of spotted owls selecting for mature and old-growth forest habitat occurs in redwood (*Sequoia sempervirens*) forests of northwestern California, where the owls frequently nest and forage in complex, naturally-regenerating early-seral forests as well [31].

The northern spotted owl was listed as a threatened species in 1990 due to extensive reduction and fragmentation of late-seral forest as a consequence of timber harvest [32]. After listing, a recovery team was established [33], but a final recovery plan was never formally adopted. Subsequently, the Northwest Forest Plan (NWFP; [22]) was developed which served as a *de facto* spotted owl recovery plan. In contrast to previous spotted owl conservation efforts [33, 34], the NWFP addressed the habitat requirements of multiple species as required by the National Forest Management Act of 1976. The NWFP more than doubled the amount of federal public land protected from intensive timber harvest within the range of the spotted owl [22].

At the time of listing, several long-term spotted owl demographic studies were initiated on federal, private, and tribal lands. Those studies have monitored vital rates and population changes over a wide portion of the species' geographic range. Since 1996, a meta-analysis of the demographic study areas’ (DSA) data has been conducted approximately every five years [35–39], each of which has reported a declining population trend overall, but with variation in vital rates and rate of population change among study areas. The analysis that coincided in time with our efforts [38] reported a mean annual rate of population decline of 2.9% range-wide during the previous >20 years, while [39] reported a range-wide decline of 3.8% per year from 1985–2013. The importance of habitat for spotted owls remains [38, 40], even though the relationship between amount of late-seral habitat and demographic rates [7], occupancy [41], and abundance [42] is not always linear.

Since the initial listing, the threat that barred owls, native to eastern North America, posed to spotted owls through interspecific competition and displacement has increased dramatically [40, 43–49]. Meta-analyses have documented negative effects of barred owls on spotted owls [38, 39]. Barred owl presence was associated with increased local spotted owl territory extinction rates on all 11 DSAs, and presence of barred owls was negatively related to spotted owl territory colonization rates in 5 of 11 DSAs [39]. Similarly, spotted owls were much less likely to use areas occurring within barred owl “core areas,” and barred owls were more numerous than spotted owls in western Oregon [50]. Overlap in habitats used by barred and spotted owls resulted in reduced territory occupancy for spotted owls, but the effect was less pronounced in areas with more high quality spotted owl habitat [51]. The barred owl’s impacts on spotted owls can be seen as a reduction of the latter’s realized niche [52] and as a stressor adding to an already challenging conservation problem.

Our modeling approach is similar to the approach proposed by others [53, 54] for designing and evaluating habitat networks, and follows the landscape conservation planning framework we outline above. We invoked four guiding principles as the basis for establishing quantitative and qualitative criteria to evaluate and compare various critical habitat networks:

1. Ensure sufficient habitat to support population viability across the range of the subspecies.
2. Support demographically stable sub-populations (see modeling regions below).
3. Ensure the distribution of spotted owl populations across their range of habitats.
4. Explicitly address sources of uncertainty (e.g., barred owl invasion, climate change, wildfire and other disturbance risk, and environmental stochasticity).

## Study area

**Fig 1:**
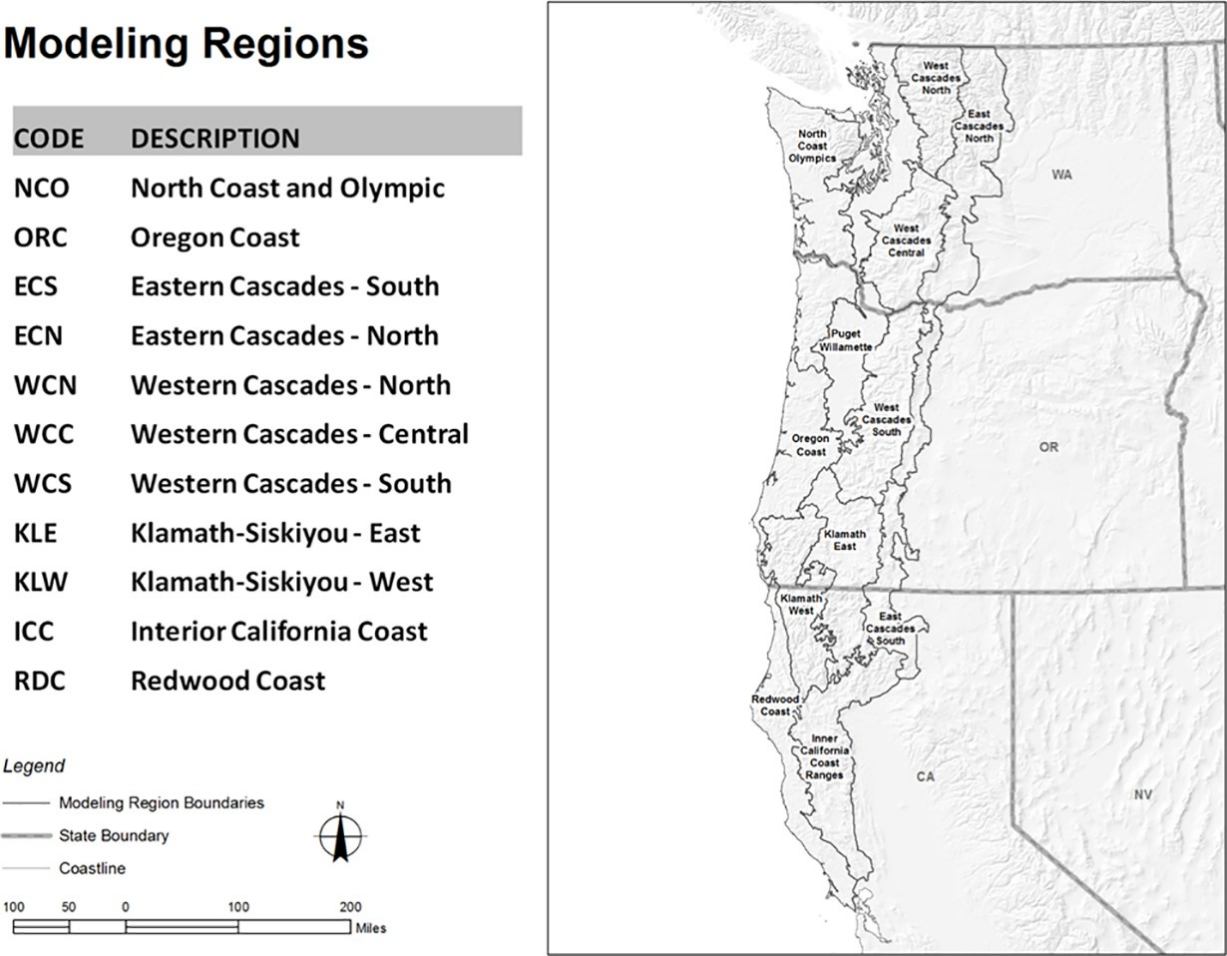
Geographic extent of study area and modeling regions boundaries.

Our study area consisted of the known range of the spotted owl in the United States (Fig 1). The spotted owl’s geographic range is 232,000 km^2^, occurs within large portions of three U.S. states (Washington, Oregon, and California) and a small portion of British Columbia, Canada, and within a variety of biophysical contexts (e.g., climate, elevation, major vegetation types). Approximately half of the lands within the study area are managed by public agencies, primarily the federal government. National Forests make up the majority (71%) of public lands in this region, followed by state owned lands (11%) Bureau of Land Management (10%), and lands managed by the National Park Service (8%) [22].

We subdivided the 232,000 km^2^ study area into 11 modeling regions that ranged in size from 10,500 km^2^ to 49,900 km^2^ (Fig 1, Table 1). These regions differ, to some extent, from previous approaches to dealing with variation among major regions within the spotted owl’s geographic range (e.g., [34]). We assumed that within each modeling region spotted owls would occur as a discrete metapopulation. More thorough descriptions of modeling regions can be found in USFWS ([45], Appendix C).

**Table 1:** Name, acronym and size of modeling regions, and definitions of acronyms used throughout.

**Modeling Region**	**Acronym**	**Size (km**^**2**^**)**
North Coast Ranges and Olympic Peninsula	NCO	49,900
Oregon Coast Ranges	OCR	17,400
Western Cascades North	WCN	12,500
Western Cascades Central	WCC	15,900
Western Cascades South of Oregon	WCS	26,100
Eastern Cascades North	ECN	26,900
Eastern Cascades South	ECS	10,500
Western Klamath	KLW	16,200
Eastern Klamath	KLE	19,700
Interior California Coast	ICC	21,300
Redwood Coast	RDC	15,600
Total		232,000
**Definition**	**Acronym**	
Area Under the Receiver Operator Curve	AUC	
Demographic Study Area	DSA	
Endangered Species Act	ESA	
Foraging habitat	F	
Generalized Nearest Neighbor	GNN	
Individual Based Model	IBM	
Nesting and Roosting habitat	NR	
Northwest Forest Plan	NWFP	
Relative Habitat Suitability	RHS	
*Strix varia*	STVA	
Zonation with all lands available	ZALL	
Zonation with priority on public lands	ZPUB	

## Materials and methods

**Fig 2:**
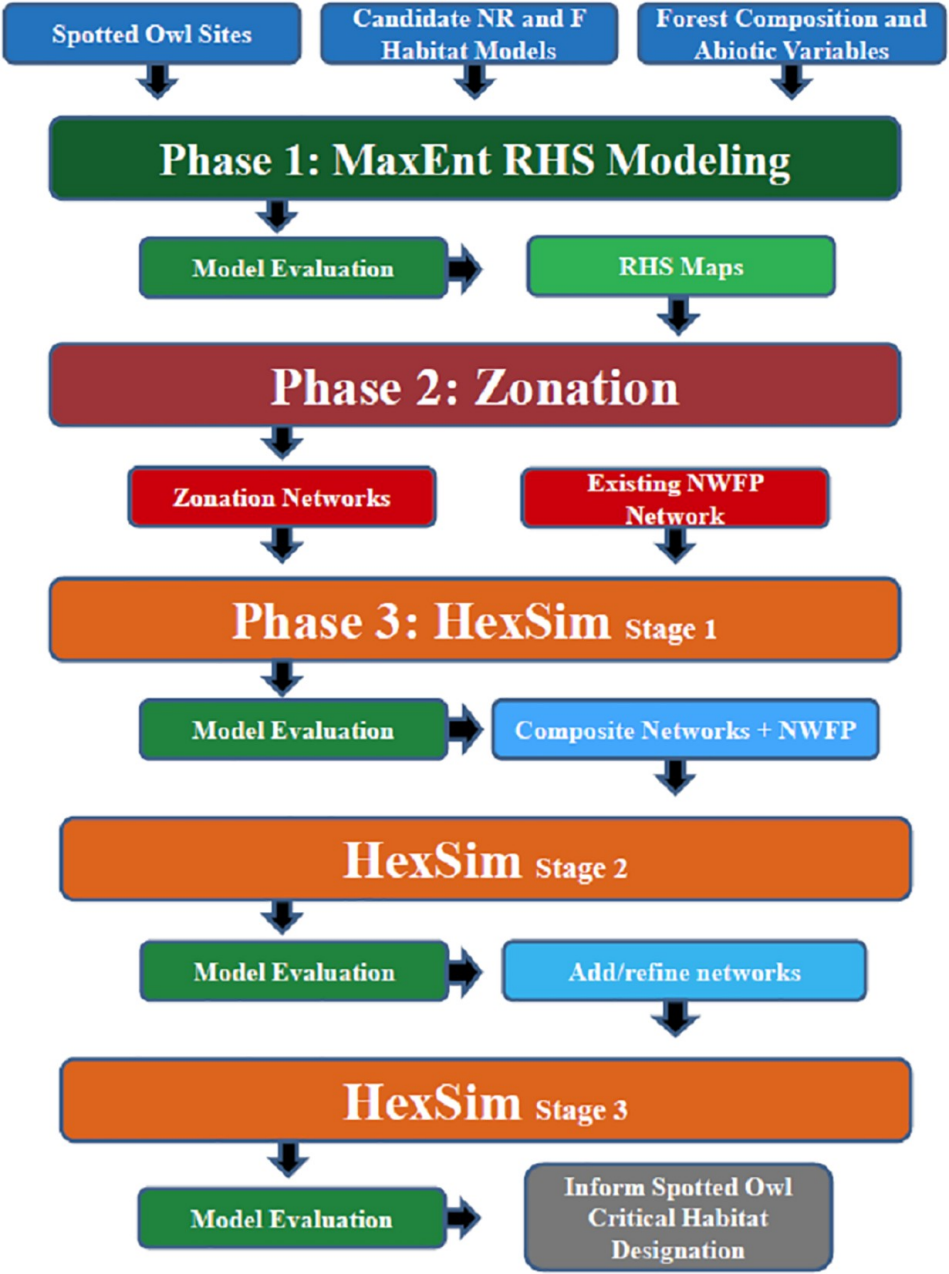
Flow chart of generalized modeling phases. NR = nesting and roosting, F = foraging, RHS = relative habitat suitability, NWFP = Northwest Forest Plan.

The modeling process involved three major analytical phases (Fig 2): 1) use of a species distribution model to estimate relative habitat suitability (RHS) in the form of a map; 2) use of the RHS base map as input to a spatially explicit conservation prioritization model to identify candidate critical habitat networks (hereafter, candidate networks); and 3) use of an individual-based, spatially explicit population model to evaluate and rank candidate networks.

### Analytical phase I: Modeling and mapping relative habitat suitability

The first phase in our modeling process (Fig 2) was to develop RHS maps for spotted owls, both at modeling regional and range-wide scales. RHS maps do not display absolute measures of habitat suitability but rather, gradients of habitat suitability relative to what is available within a given modeling area. We modeled RHS for individual modeling regions based on evidence that the habitat relationships of spotted owls vary geographically [20]. Because much of the spotted owl location data used in our modeling was presence-only data, we used MaxEnt [55] to model RHS. MaxEnt was developed specifically for presence-only data [55], has been extensively tested on many datasets and species, and was found to perform better than most other modeling tools [56]. We used a variety of metrics to evaluate RHS models and maps before incorporating them into the second phase in our modeling process aimed at identifying candidate networks (see Analytic phase II: identifying candidate potential critical habitat networks, below).

### Data for relative habitat suitability modeling

We modeled RHS using spotted owl site centers, which are the surveyed locations of nests or daytime roosts used by pairs. We used site center data from spotted owl DSAs, the NWFP Effectiveness Monitoring Program [57], and other sources. RHS models were created at the 200-ha scale (see below).

Barred owls have displaced many spotted owls from previously occupied nesting areas, sometimes into habitat types or conditions that spotted owls rarely used prior to the barred owl’s invasion [44, 58]. To develop RHS models for spotted owls with a focus on habitats selected prior to extensive barred owl influence, we used vegetation data from 1996 (see below). We followed previous research [57] and restricted our spotted owl location data to site centers from surveys during 1993–1999 so that our owl locations and vegetation data were from roughly the same time period (see S1 Appendix). We also thinned site center locations to avoid sample selection bias ([59]; see S1 Appendix for details).

We used 23 biotic variables (see S1 Appendix) from Gradient Nearest Neighbor (GNN; [60]) vegetation data developed for the NWFP’s Effectiveness Monitoring Program [61, 62]. We used GNN because it provided detailed maps of forest composition and structural attributes for all lands within the NWFP area [63] and was the sole “wall-to-wall” vegetation map for the entire study area. For our modeling, we selected 23 biotic variables (Table 2) from a set of 163 GNN output variables. The reliability or accuracy of vegetation databases poses a primary concern for wildlife habitat evaluation and modeling [64], but pixel-level inaccuracies of the GNN data are less influential when applied at larger scales [65].

**Table 2:** List of variables used for modeling relative habitat suitability of northern spotted owl site centers.

**Forest Structure**	**Definition**
CANCOV	Canopy cover of all live trees
CANCOV_CON	Canopy cover of all conifers
DDI	Diameter diversity index (structural diversity within a stand, based on tree densities within different DBH classes)
SDDBH	Standard deviation of DBH of all live trees
MNDBHBA_CON	Basal area weighted mean diameter of all live conifers
TPH_GE_50	Live trees per hectare greater than or equal to 50 cm DBH
TPHC_GE_50	Conifers per hectare greater than or equal to 50 cm DBH
TPH_GE_75	Live trees per hectare greater than or equal to 75 cm DBH
TPHC_GE_75	Conifers per hectare greater than or equal to 75 cm DBH
TPHC_GE_100	Conifers per hectare greater than or equal to 100 cm DBH
QMDC_DOM	Quadratic mean diameter of all dominant and co-dominant conifers
BAA_GE_3	Basal area of all live trees greater than or equal to 2.5 cm DBH
BAA_3_25	Basal area of all live trees 2.5 to 25 cm DBH
BAA_GE_75	Basal area of all live trees greater than or equal to 75 cm DBH
BAC_GE_3	Basal area of conifers greater than or equal to 2.5 cm DBH
BAC_GE_50	Basal area of conifers greater than or equal to 50 cm DBH
BAH_PROP	Proportion of BAA_GE_3 that is hardwood
BAH_3_25	Basal area of all live hardwoods 2.5 to 25 cm DBH
**Forest Composition**	**Definition**
Evergreen Hardwoods	Basal area of tanoak, canyon, coast and interior live oaks, giant chinquapin, California bay and Pacific madrone
Subalpine	Basal area of silver fir, mountain hemlock, subalpine fir, red fir, Engelmann spruce,
Pine	Basal area of ponderosa pine, Jeffrey pine, lodgepole pine, and Bishop pine
Northern Hardwoods	Basal area of red alder and bigleaf maple
Oak Woodland	Oregon white oak and blue oak

Spotted owl habitat is often classified into dispersal, foraging, roosting, and nesting habitat (e.g., [45]), with each being a consecutive superset of the previous type(s). Habitats used for nesting and roosting are very similar [34], which we combined into nesting-roosting. In our modeling, we attempted to map the suitability of habitats used for breeding by spotted owls. Thus, we evaluated and modeled nesting-roosting and foraging habitat, but not dispersal habitat. Using various combinations of the forest structure variables (Table 2), we developed a suite of nesting-roosting (NR) and foraging (F) habitat definitions specific to conditions in each modeling region (see Appendix C in [45]). Each 30x30 m pixel was either NR, F, or neither habitat type, and the total amount of NR and F within a 200-ha (800-m radius) area around each pixel was estimated.

The spatial arrangement of habitat, particularly the amount of habitat patch "edge" and "core" (i.e., patch interior), influences both habitat selection and fitness of spotted owls within the southern portion of their range [7, 66]. Therefore, we also estimated NR core and edge. NR core was estimated as the total area of habitat within a 200-ha circle surrounding each pixel remaining after each NR patch was internally buffered by three pixels around its entire perimeter (i.e., 90 m), and the buffered area subtracted from the total area. Edge was likewise estimated within the 200-ha circle surrounding each pixel and was the total length of NR habitat patch perimeter wherever it was adjacent to non-NR habitat.

We evaluated eight abiotic features known or suspected to influence spotted owl habitat selection and use (Table 3). Local geographic features such as slope position, aspect, distance to water, and elevation influence spotted owl NR site selection (e.g., [30, 67, 68]). Several authors noted the absence of spotted owls above elevation limits that varied geographically (e.g., [69–71]). At broader spatial scales, temporal variation in climate has been related to fitness [7–9, 72], suggesting that spatial variation in climate may also influence habitat suitability for spotted owls. Mexican spotted owls (*S*. *o*. *lucida*) have a narrow thermal neutral zone [73] and it has been assumed [7] that the northern spotted owl is similar in this regard. Furthermore, the spotted owl’s selection for areas with older-forest characteristics has been hypothesized to, in part, be related to its needing cooler areas in summer to avoid heat stress [74]. Temperature extremes (winter low and summer high) as well as potential breeding-season specific stressors (spring low temperature and high spring precipitation) were also included as candidate predictor variables in our models [72, 75].

**Table 3:** Categories of candidate variables (and order of entry), variable names of variables used in the relative habitat suitability modeling process.

Category	Variable
Best climate/elevation model (5)	Mean July Precipitation
	Mean July Temperature
	Mean July Precipitation
	Mean July Temperature
	Mean Elevation
Topographic position (4)	Curvature
	Insolation
	Slope Position
Compositional variables (percent of basal area; 3)	Redwood
	Oak Woodland
	Pine-dominated
	Northern Deciduous Hardwoods
	Evergreen Hardwoods
	Douglas-fir
	Subalpine forest
Habitat pattern (2)	Core of NR habitat
	Edge of NR habitat
Habitat structure (1)	Foraging Habitat Amount
	Nesting/Roosting Habitat

Some forest types could have structural attributes suitable for spotted owls (e.g., high canopy cover, multi-layered canopy, large trees) and yet be rarely used by the species (e.g., ponderosa pine (*Pinus ponderosa*): [76]). We attempted to account for this possibility by evaluating models that included dominant tree species composition (amount within 200-ha of site centers) variables (Table 3).

To determine the spatial scale at which to develop RHS models, we adopted a uniform analysis area size that corresponded to large differences between use and availability at the individual owl territory scale, while also considering the spatial scale at which the GNN data could be reliably used. Studies of spotted owl habitat selection have reported differences between spotted owl-centered (nest or activity center) locations and random or unoccupied locations across the range of spatial scales examined (e.g., [31, 41, 77–80]). However, the largest differences were often found in areas approximately the size of what was defined as “core areas” [81]; that is, areas of the home range that receive disproportionate use, and we recognize that core area alone is likely insufficient to provide for the species [82]. Based on an extensive review of published studies [7–9, 41, 57, 79, 80, 83–85], we chose a 200-ha analysis area centered on spotted owl site centers to represent core areas. We assumed that our modeled core areas approximated individual spotted owl territories. Geographic variation in home range size was explicitly addressed at phase three in our modeling.

There is higher uncertainty associated with GNN data at smaller spatial extents than at larger extents [64]. Although the GNN data are available at 30-m resolution, our RHS models were developed at 200 ha, and applied to the 11 modeling regions. Hence, each pixel’s RHS value was a function of conditions within an 800-m radius (200 ha) of it (but other pixels in that 200-ha area had different RHS values, based on the conditions within 800-m around them). Thus, our smallest extent of evaluation is a modeling region which is consistent with previous recommendations [60] that GNN maps are appropriately used for regional-level planning, rather than local management decisions.

### Developing relative habitat suitability models

To estimate RHS, we used MaxEnt [55] to compare the characteristics (variables included in the models) of training data sites (spotted owl site centers) to a random selection of approximately 10,000 “background” (available) locations. We used linear, quadratic, and threshold covariate features within MaxEnt. We rescaled the logistic output of MaxEnt to range from 0 to 100. We masked (removed from consideration as habitat) areas above elevations used by spotted owls in each modeling region as well as all non-potential habitat (e.g., lakes, cities, non-forested areas, agricultural areas).

**Fig 3:**
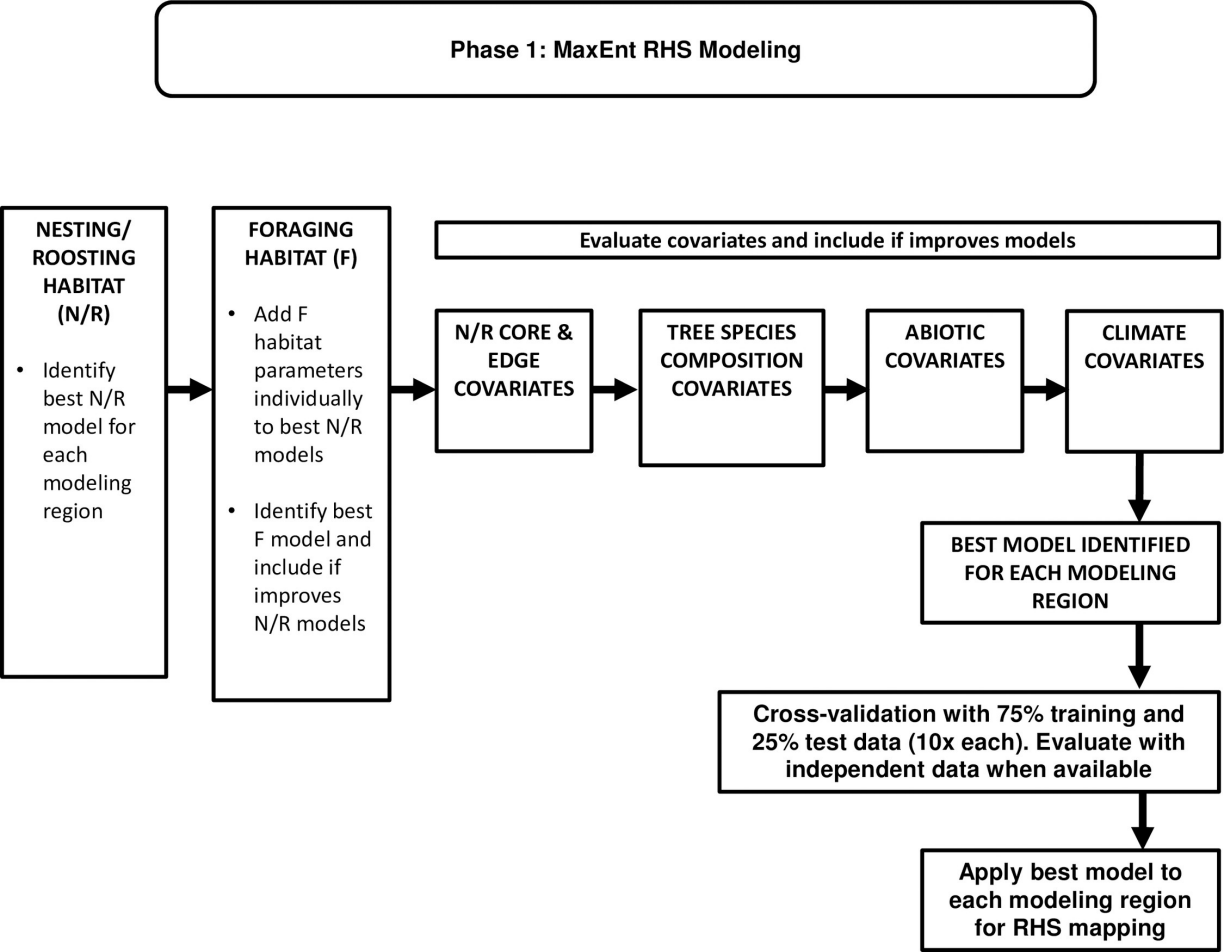
Flowchart of development and evaluation of MaxEnt relative habitat suitability (RHS) models within each of 11 modeling regions.

We used a model-building process (Fig 3) to determine the best model for each modeling region. At each step, the best performing model at that point (hereafter, best model) was “challenged” by adding other variables or combinations of variables to it (*sensu* [86]), in an attempt to improve its predictive ability. Models were evaluated based on the average rank of their area under the receiver operating characteristic curve (AUC) and gain ([14]; see below).

### Evaluating relative habitat suitability models

We sought models that had good discriminatory power, were well calibrated, were robust, and had broad generality. We evaluated model discrimination via AUC. We assessed model calibration by comparing ranks of area adjusted frequencies (*sensu* [87]) to RHS bin rank. Robust models are those that perform well under cross-validation and are not simply well-fit to the full training data set (i.e., they are robust to perturbations of the developmental data set). We evaluated model generality using independent data when it was available. A model with good generality performs well on independent data. MaxEnt balances model fit and complexity through the use of regularization [88]. MaxEnt fits a penalized maximum likelihood model [88], closely related to other penalties for complexity such as Akaike’s Information Criterion [89]. To evaluate whether any model region’s model was over-fit, we conducted cross-validation on each model, and, when possible, we evaluated how well models classified independent data (Fig 3).

Model discrimination and model calibration are independent measures [90]. Model calibration reflects the agreement between proportion of the modeling region in each RHS bin and observed proportions of owl sites within RHS bins [90, 91]. A “continuous Boyce index” for species distribution models uses a moving-window approach [92], which we refer to as “strength of selection” (SOS). SOS assesses the distribution of presence locations (use) by RHS bins relative to the abundance of such areas in the modeling regions (availability). A well-calibrated model will show the species to use higher suitability areas disproportionately more and lower suitability areas disproportionately less than expected based on area.

Using each modeling region’s best model, we conducted cross-validation to evaluate model fit and how robust the model was. Each of 10 times, we removed a random subset of 25% of the spotted owl site centers, developed the model with the remaining 75% (training data) and classified the withheld 25% (test data). AUC was evaluated for both training and test data. The difference between training and test AUC values indicates the extent to which models are over-fit [93], because over-fit models generally have high calibration accuracy and perform poorly on data not used in the model’s development. We evaluated the difference in AUC values between training and cross-validated (test) models.

We had samples of independent spotted owl site centers gathered from 2003 to 2009 and compared their RHS values to corresponding values for spotted owl site centers used in model development. All test sites were more than 0.8 km from any training site. Comparison with independent spotted owl site centers from 2006 enabled us to evaluate accuracy of the models when projected to a new time period (model transferability), and to investigate systematic shifts in RHS at spotted owl sites. These shifts may occur, for example, in areas where densities of barred owls increased during 1996–2006 and displaced spotted owls from favorable habitat. If this is the case we would expect to see reduced use of high RHS areas at 2006 spotted owl sites, relative to 1996 values.

### Analytical phase II: Identifying candidate networks (Zonation)

In order to identify candidate critical habitat networks, we used each modeling region’s best RHS model as a single species file, the biodiversity feature, to input into the conservation planning model Zonation [94]. The RHS files were comprised of estimates of RHS for each 30-m pixel (based on the 200-ha around them) within each modeling region, and therefore, throughout the spotted owl’s geographic range. Zonation produces a hierarchical prioritization of the landscape based on the habitat value of cells [95]. In our application, a cell is a 30-m pixel and its habitat value was a function of its RHS value and the values of neighboring pixels. The resulting solution is hierarchical, meaning that the most valuable five percent of habitat is retained within the most valuable 10 percent; the most valuable two percent is retained within the valuable five percent, etc. We used the Core Area Zonation cell removal function, and added 200 edge points per modeling region. Within Zonation we also used an α-value of 0.000952 for distribution smoothing [94], based on a buffer distance around each pixel of 2.1 km (to approximate a home range sized area).

We conducted Zonation analyses separately within each modeling region to reflect geographic variation in the spotted owl's habitat relationships and to attempt to meet the goal that spotted owls and critical habitat would be well-distributed throughout the species' range. Zonation allows for the identification of any percentage of habitat value to display as maps of candidate networks. Selection of the percentage of habitat value has a large influence on the size and distribution of networks. We evaluated the amount of area and abundance of various RHS classes within a broad range of Zonation-defined habitat values (30%, 50%, and 70%), with the objective of identifying a smaller subset of diverse (e.g., varying size and ownership) candidate networks for testing with the individual-based model.

**Fig 4:**
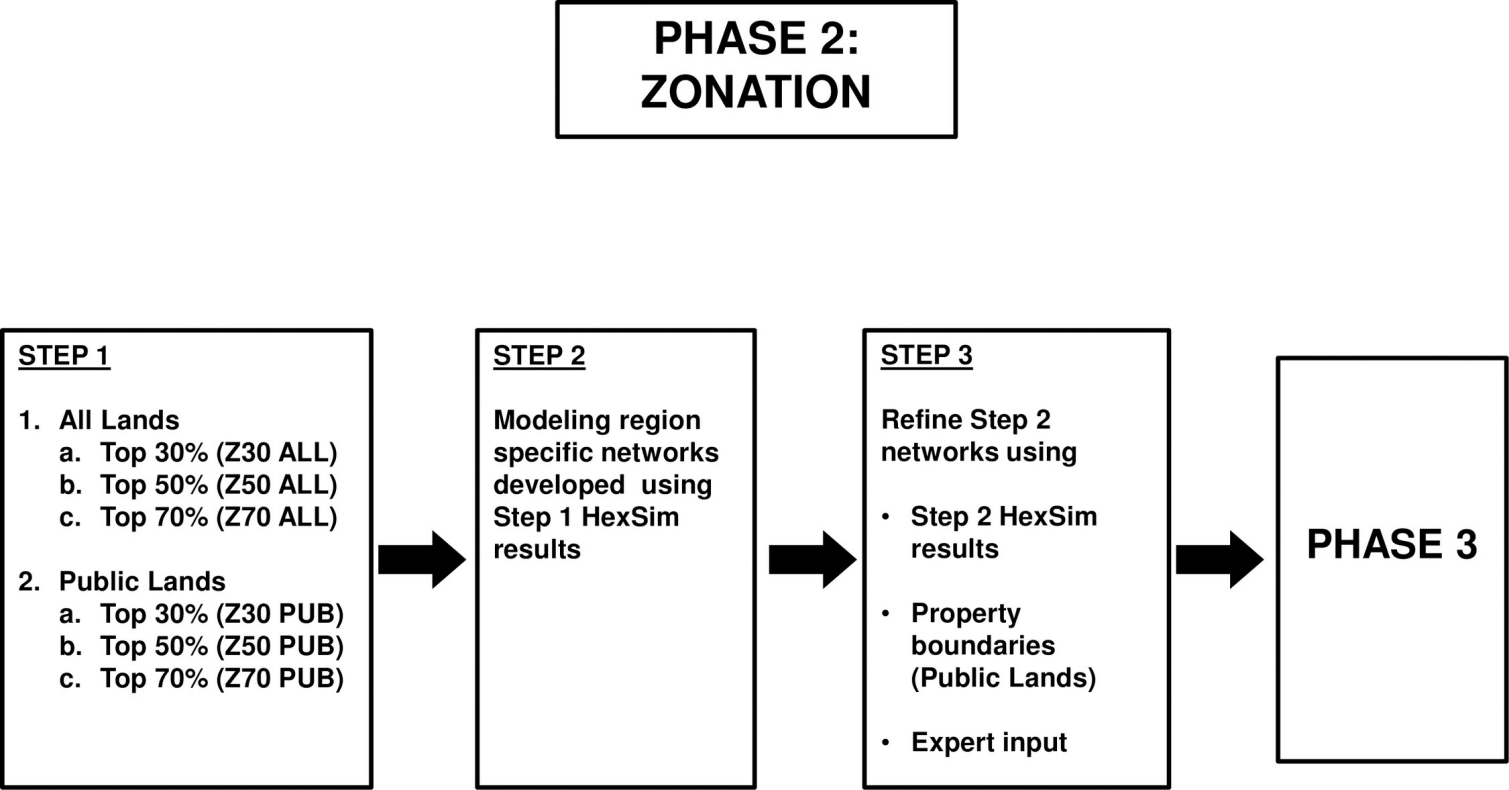
Flowchart of steps using Zonation to identify alternative candidate critical habitat networks for northern spotted owls.

We developed 18 candidate Zonation-defined networks, expressed in terms of area of various RHS classes (Fig 4), to existing networks including previous spotted owl critical habitat designations (1992 and 2008) and the NWFP reserve network. We did not force the inclusion of previously existing public lands with special protection (e.g., Wilderness Areas or State Parks) in any networks, but we did assume that such areas would continue to provide habitat in the future (see below). We then evaluated simulated spotted owl population outcomes within and among each of those networks (see below).

### Analytical phase III: Evaluating candidate networks using individual-based spatially explicit population modeling

Whereas other approaches such as population viability analysis (PVA) and meta-population models have been used for evaluating spotted owl populations (e.g., [96]), we required an approach that enabled comparison of a wide range of spatially explicit conditions such as variation in size and spacing of candidate critical habitat networks, and varying RHS and barred owl impacts over time. Spatially-explicit individual-based models (IBMs) allow for the representation of ecological systems in a manner consistent with the way ecologists view such systems as operating.

IBMs need to be simple enough to be practical, but have enough resolution to capture essential structures and processes [97]. For the spotted owl there exists a large quantity and quality of data on: vital rates from several long-term DSAs [37–39], habitat selection (see review by [21]), and dispersal [98], among many other aspects of the species’ ecology, and it is thus well-suited for spatially-explicit IBM. IBMs allowed us to conduct “model experiments” that would be impossible with live animals [97] and real landscapes.

HexSim [99] is an IBM designed for simulating terrestrial wildlife population dynamics and interactions. HexSim was designed to quantify the cumulative impacts to wildlife populations of multiple interacting stressors. HexSim was used to evaluate effects of size and spacing of spotted owl habitat areas on owl population size and trend [100]. Sensitivity analysis subsequently established HexSim as a viable tool for modeling spotted owl populations [101]. We developed a HexSim spotted owl scenario (see [102] for details) based on the most up-to-date demographic data available on spotted owls [38], published information on spotted owl dispersal [98], and home range size [21], as well as on parameters for which less empirical information was available. HexSim serves as a consistent framework into which variation in spatial data layers (*e*.*g*., candidate network sizes and distributions; different assumptions about habitat conditions (RHS) inside and outside of networks; different assumptions about RHS change on public versus private lands; and different assumptions about the impact of barred owls among modeling regions) can be introduced. Comparison of estimates of simulated spotted owl population performance across the range of scenarios incorporating variation in network sizes, RHS trends, and barred owl influence, can provide insights about candidate networks and other conservation measures designed to lead to spotted owl recovery.

Each HexSim simulation run provides estimates of population size (breeders and non-breeding floaters) at any chosen time step as well as population trend over any range of time steps. Estimates are reported at both range-wide and modeling region scales. The results are intended to allow comparison of *relative population performance* among networks, not precise predictions of actual population size in the future.

When a HexSim simulation starts, the number of individuals, age class distribution, spatial arrangement of territories, and other population attributes will have values that reflect the model's initial conditions. It takes many time steps (“model years”) for these artifacts to subside past the start-up bias period, and thus for the population's stable-state dynamics to become evident. We started simulations with 10,000 female spotted owls, thus this initial period of transitory dynamics involved a period of rapid population decline for the first 25 or 30 time steps ("free response" analysis [102]); typically subsiding by approximately time step 50 [102]. It is important not to confuse this decline with an observed or predicted loss in spotted owl numbers that has resulted from changing environmental conditions.

#### Overview of the spotted owl scenario in HexSim

**Fig 5:**
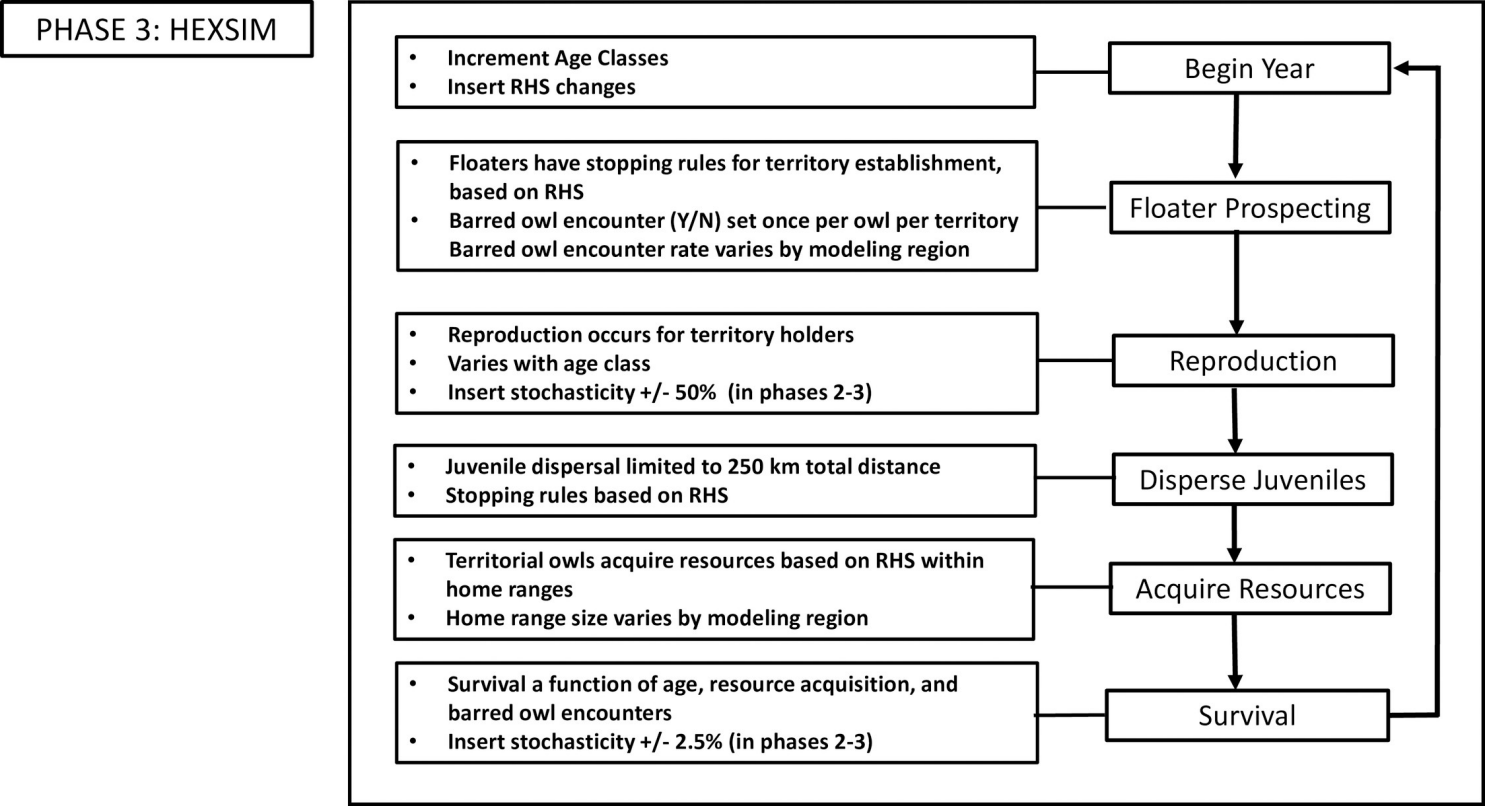
Flowchart of generalized steps involved in the northern spotted owl HexSim model.

Here, we reference the northern spotted owl life history simulator documented in [102]. In summary, this is a spatial individual-based, females-only model, because female owls are the most influential sex in terms of population dynamics [103]. Individual vital rates and movement decisions are influenced by the RHS habitat map described above, by variation in home range size among modeling regions [104], by resource acquisition rates capturing landscape structure and conspecific competition, stage class, and by the spatially-inferred presence of competition from barred owls (Fig 5). The model has been used to forecast both range-wide and regional population trends, and to quantify emergent movement patterns and demographic source-sink dynamics [102].

The HexSim spotted owl simulator was purposely kept parsimonious and defensible, in part due to anticipated legal challenges to the recovery plan [45] and designation of critical habitat [105] it informed. Future applications of the model could be improved if two key enhancements were made. First, underlying habitat map could be updated and perhaps made dynamic, so as to include ongoing land use, climate change, and altered fire regimes. Second, a full two-species version of the model could be developed that enabled the direct simulation of northern spotted owl and barred owl interactions. Work towards this latter goal is ongoing (David Wiens, pers. com.). Details of the northern spotted owl HexSim model can be found the S1 Appendix.

#### Evaluation of HexSim model calibration

We compared simulated spotted owl population sizes in eight DSAs to empirically estimated number of spotted owls on those DSAs to evaluate the model’s ability to accurately predict owl numbers. For calibration purposes data from the eight DSAs were used. We subsequently tuned various parameters in the model (e.g., resource acquisition target, home range size, and dispersal) to reduce differences between simulated and empirical estimates. The numbers of female spotted owls were tracked range-wide, per modeling region, and per DSA. We compared simulation time step-50 HexSim estimates to field data from the DSAs. For these comparisons, we used the HexSim simulations during which barred owl impacts were inserted during time step 40 and remained constant for the remaining time steps. We used a static RHS map for these initial evaluations.

HexSim simulations are stochastic, and to quantify population size for Phase 1 simulations, the mean was taken from five replicate simulations. Although five replicates was relatively small, at this stage of modeling we were interested in fairly coarse-scale questions such as whether simulated population sizes were close to real-world population sizes. Each simulation was 250 time steps in duration. The length of the simulations (250 time steps) allowed a steady-state population size and trend to be estimated.

Dispersal is a critical process through which landscape structure affects spotted owl population size and metapopulation structure, and is a primary concern in conservation network design [15]. Of particular importance is natal dispersal. We evaluated natal dispersal distances of simulated owls in HexSim relative to empirical estimates of dispersal distances of juvenile spotted owls [98]. The dispersal behavior of the simulated spotted owls was affected principally by landscape structure, the dispersal stopping criteria, and the degree of autocorrelation of movement direction.

#### HexSim sensitivity analyses

We conducted sensitivity analyses of the spotted owl HexSim model (*sensu* [101]) by modifying nine separate parameter values. Seven of the nine were subjected to two modifications (one decrease and one increase), one was assigned four distinct values (two lower and two higher), and one was modified three times (S1 Appendix).

#### Development of scenarios for evaluation and comparison in HexSim

We simulated spotted owl population performance relative to three primary sources of variation: overall size (area) and distribution of networks; differences in amount and quality of RHS inside and outside of candidate critical habitat networks; and the influence of barred owls. Considering the many possible variations in network designs, land ownership limitations, future habitat trends, and barred owl effects that could be evaluated, it is clear the number of scenarios needed to evaluate all of the possibilities could increase rapidly and become unfeasible. Instead, we developed an iterative process for evaluation of scenarios, which ranged from a series of relatively simple, coarse-scale, evaluations (Phase 1) to more complex, finer-scale evaluations (Phases 2 and 3).

We created RHS change scenarios to represent variation in habitat management within and outside of candidate critical habitat networks (i.e., one would expect areas of critical habitat to be managed different than areas not in critical habitat). Zonation-generated networks were developed with only two simple precedence masking rules: (1) no land-ownership restrictions (ZALL networks) and (2) prioritizing public lands before considering the inclusion on non-public lands (ZPUB networks). We treated previously existing protected public lands similar to those lands selected by Zonation. Previously existing protected public lands included all Congressionally Reserved lands (i.e., National Parks and Wilderness Areas), NWFP Late Successional Reserves, and State Park lands.

For Stage1 simulations, we compared spotted owl population responses among six candidate networks derived from Zonation plus the NWFP (Table 4), three RHS change scenarios, and four barred owl scenarios, for a total of 84 network by scenario combinations. These enabled us to evaluate relative performance among simulated spotted owl populations within individual modeling regions and range-wide. We considered Stage1 simulations coarse-scale and used them to inform the creation of Stage2 networks.

**Table 4:** Number of alternative candidate critical habitat networks by relative habitat suitability by barred owl scenarios, inclusion of environmental stochasticity, number of replicates, and total time steps used in HexSim simulations of northern spotted owl populations.

Phase (number of networks)	Relative Habitat Suitability change scenario	Barred Owl scenario	Environmental Stochasticity Included	Number of replicates	Simulation time-steps
1 (7)	HAB1	STVA1	no	5	250
	HAB1	STVA2	no	5	250
	HAB1	STVA3	no	5	250
	HAB1	STVA4	no	5	250
	HAB2	STVA1	no	5	250
	HAB2	STVA2	no	5	250
	HAB2	STVA3	no	5	250
	HAB2	STVA4	no	5	250
	HAB3	STVA1	no	5	250
	HAB3	STVA2	no	5	250
	HAB3	STVA3	no	5	250
	HAB3	STVA4	no	5	250
2 (3)	Optimistic	STVA5	yes	100	350
	HAB1	STVA5	yes	100	350
3 (9)	Optimistic	STVA5	yes	100	350
	HAB1	STVA5	yes	100	350

We evaluated three RHS change scenarios for Stage1 modeling. HAB1 consisted of maintaining RHS values within networks at their currently-estimated values, and reducing all non-network lands with RHS values ≥35 to a value of 34. This scenario was intended to simulate an “isolated” network by only allowing territory establishment within networks. In HexSim, territory establishment was only allowed to happen when hexagon RHS values were ≥35 for three adjacent hexagons (S1 Appendix). Areas outside of networks could still be incorporated into simulated home ranges and thereby contribute resources to owls, but territories were restricted to networks in this scenario. In scenario HAB2, we maintained the RHS value within networks at their current estimated values, and reduced all non-network areas with RHS values ≥35 to a value of 34, but maintained RHS >50 on non-network areas on *public lands* at their currently-estimated values. This scenario was intended to emulate the management approach of maintaining occupied spotted owl habitat outside of networks (e.g., full implementation of Recovery Action 10 [45]) on *public lands*. Scenario HAB3 was identical to HAB2, except that RHS >50 was maintained on all non-network lands, regardless of ownership. This scenario simulated full implementation of Recovery Action 10 [45] on *all lands*. For the purposes of developing RHS scenarios in Stage1, Congressionally Reserved lands (e.g., Wilderness Areas and National Parks) were treated as part of networks, regardless of whether Zonation had selected these areas. This was done because such areas were set aside by acts of Congress, and we assumed that RHS would be retained.

Barred owl scenarios used for Stage1 included: STVA1 in which we assumed no barred owls existed (i.e., barred owl encounter probability was set to zero for all individual spotted owls in all places); STVA2 in which barred owl encounter probabilities were held constant at their current estimated rates within each of the 11 modeling regions; STVA3 in which barred owl encounter probabilities were held constant at 0.25 everywhere in the spotted owl’s range; and STVA4 in which barred owl encounter probabilities were held constant at 0.5 everywhere in the spotted owl’s range. For Stage1 simulations, barred owl encounter probabilities were inserted at time step 40, and RHS changes were inserted at time step 50. Population performance metrics were evaluated range-wide and for each modeling region.

The following range-wide population performance metrics were used to compare and rank the Stage1 networks by habitat and barred owl scenarios: 1) mean percentage population change among the five replicates between time steps 50 and 250; 2) percentage of time steps during which population growth rate (λ; mean of five replicates ± 95% CI) was ≥1.0 between time steps 50 and 250; and 3) the first year that λ (mean ± 95% CI) was ≥1.0. Because we were interested in longer-term trends, we calculated λ as N_t_/N_t-10_ rather than by successive time steps.

For each modeling region we evaluated: 1) percentage of time steps during which λ (mean of five replicates ± 95% CI) was ≥1.0 between time steps 50 and 250; 2) the first year that λ (mean ± 95% CI) was ≥1.0; 3) the percentage of replicates during which the population fell below 250 individuals; 4) the percentage of replicates during which the population fell below 100 individuals; and 5) the percentage of replicates during which the population went to extinction. The thresholds of 250 and 100 individuals were considered to be quasi-extinction thresholds (i.e., population sizes that we believed to be at relatively high risk of extinction). By not including environmental stochasticity in Stage1 simulations, simulation results were more likely to provide optimistic results (i.e., predisposed to underestimate extinction risk). Thus, we chose the two quasi-extinction thresholds.

Stage1 simulations were intended to provide coarse-level information that would be used to refine and create subsequent networks which would be subjected to more thorough evaluations. Therefore, the evaluation of Stage1 simulations included comparing the quantitative measures articulated above, as well as our professional judgment. For example, we carefully considered the fact that only five replicates were run for each of the 84 combinations of network by RHS change by barred owl scenarios in Stage1. We generally ignored small differences in the performance of networks relative to our evaluation metrics. Furthermore, we did not weigh each performance metric equally. For example, one of the 84 combinations might have had a population that was the first to achieve population stability (λ ≥ 1.0), but subsequently declined rapidly or became very unstable over the longer-term. Longer-term stability was considered more important in such circumstances.

Stage2 simulations were developed after evaluating the performance of Stage1 simulations. Stage2 simulations were run on three networks, two of which were created based on the results of Stage1 (Composite 1 and Composite 2), and the NWFP. Composites 1 and 2 were considered to be of lower and higher risk to spotted owls, respectively. Composites 1 and 2 (see Table 5), and all subsequent Composites (see below), were not “one-size-fits-all” (e.g., Z50ALL in all modeling regions) as were the Zonation-derived networks in Stage1. Instead we evaluated the performance of previous networks within individual modeling regions and refined range-wide networks on a modeling region by modeling region basis, as described below.

**Table 5:** Candidate critical habitat networks Composites 1 and 2 that resulted from Phase 1 modeling.

		Habitat Network Scenario		
Modeling region	Barred owl encounter rate for HexSim models after Phase 1 (currently estimated encounter rate)	Composite 1 (lower risk)	Composite 2 (higher risk)	NWFP
OCR	0.375 (0.710)	Z50Pub	NWFP+Elliott State Forest	NWFP
KLW	0.25 (0.315)	Z50Pub	Z30Pub	NWFP
RDC	0.25 (0.205)	Z30Pub+HCPs	All public lands	NWFP
KLE	0.25 (0.245)	Z50Pub	Z30Pub	NWFP
ICC	0.25 (0.213)	Z50Pub	Z30Pub	NWFP
WCS	0.375 (0.364)	Z50Pub	Z30Pub	NWFP
WCC	0.375 (0.320)	Z70Pub	Z50Pub	NWFP
WCN	0.375 (0.320)	Z70Pub	Z50Pub	NWFP
NCO	0.375 (0.505)	Z70PUB—with addition of SOSEAs^/1^ plus Satsop stepping stone^/2^ (private land). RHS artificially inflated to = 0.4 at step 1 within Satsop but not SOSEAs	NWFP with the addition of Satsop, Capitol State Forest, Lower Chehalis, and SOSEAs. RHS artificially inflated to = 40 at step 1 within all additions except SOSEAs.	NWFP
ECN	0.375 (0.296)	Z70all	Z70Pub	NWFP
ECS	0.25 (0.180)	Z70Pub	Z50Pub	NWFP

^/1^: SOSEA (Spotted Owl Special Emphasis Areas) are geographic areas as mapped in Washington State's Forest Practices Rules (WAC 222-16-086). Each delimited SOSEA polygon contains the specified goal for that area to provide for demographic and/or dispersal support as necessary to complement the northern spotted owl protection strategies on federal land within or adjacent to the SOSEA. These are private lands that have special protections for owl circles.

^/2^: “Satsop stepping stone”–a portion of the Satsop River watershed selected for evaluation of population response to increased connectivity that would potentially be provided by the inclusion of this area.

Composite networks were made up of modeling region-specific Zonation or NWFP networks based on how simulated northern spotted owl populations performed in those networks. These composite networks include both the modeling region-specific habitat network scenario from Phase 1 as well as the assumed barred owl encounter rate for Phase 2–3 modeling. Composite 1 was considered to be lower risk and Composite 2 was considered higher risk. The NWFP column shows that all networks that we evaluated were always compared to the NWFP.

For Stage3 simulations we used identical comparisons, both range-wide and within modeling regions, as Stage2. Stage3 simulations included nine additional candidate networks, Composites 3–11. Composites 3–11 were either successive refinements of earlier networks or recommended by federal land management agencies (Composite 5). Efficiency of candidate networks was evaluated by comparing network size to spotted owl population performance metrics. Given similar performance, smaller networks (more efficient networks) are preferred.

We evaluated the influence of two scenarios of RHS change on simulated spotted owl populations in Stages2 and 3. Our goal was to evaluate *relative* population performance among a range of candidate critical habitat networks, so we developed two contrasting scenarios that directly projected RHS values into future conditions. The two RHS change scenarios used in Stages2 and 3 were dubbed “optimistic” and “pessimistic.”

These RHS change scenarios were not intended to be predictions, forecasts, or recommendations of future habitat conditions. The goal of these scenarios was to evaluate how different the various spotted owl population outcomes were as a function of different RHS change scenarios, not to estimate size of spotted owl populations under expected or predicted conditions. We chose the optimistic and pessimistic scenarios to reflect plausible futures, not the most extreme best- and worst-cases we could imagine. The optimistic scenario was an attempt to model future habitat that was maintained *not only* in protected areas, but was fairly well distributed throughout the landscape. In contrast, the pessimistic scenario was an attempt to model future habitat that was concentrated within candidate critical habitat networks.

For the *optimistic scenario* we used estimates of RHS change that were measured between 1996 and 2006, and projected these rates into the future (S1 Appendix). Because the primary goal of these evaluations was to compare simulated spotted owl population performance across a range of candidate networks, the objective of the *pessimistic scenario* was to simulate isolated networks by increasing contrast between network and non-network areas. The pessimistic scenario used in Stage2 and 3 modeling was identical to HAB1 scenario used in Stage1. In this scenario we held RHS *within* networks constant at its 2006 estimated level, whereas *outside* of networks we truncated all RHS values that were ≥35 to a value of 34; just below the value needed for territory establishment. All other non-network areas (already <35) remained constant. This generalized pessimistic scenario did not reflect a plausible scenario for the RDC modeling region, where privately owned lands continue to support large numbers of spotted owls despite a long history of intensive timber management. Thus, for the RDC *pessimistic scenario*, RHS *within a network* remained constant at its estimated 2006 level, whereas RHS *outside of a network* was reduced by 5% in each of two 20-year time steps. During Stages2 and 3, for private lands on which landowners had either Habitat Conservation Plans or Safe Harbor Agreements (www.fws.gov/endangered/) that would be anticipated to maintain some level of habitat, we held RHS values at their 2006 estimated levels under both optimistic and pessimistic RHS scenarios. This assumption resulted in these private lands being managed intermediately between network lands and non-network lands.

For HexSim modeling in Stages2 and 3, we used a constant barred owl encounter rate within, but not among, modeling regions (see Table 5). Stage1 modeling revealed the strong impact that barred owl encounter rate had on population performance metrics regardless of trends in RHS. In Stage1, modeling regions with high barred owl encounter probabilities, particularly in Washington and coastal Oregon spotted owl populations declined rapidly under all RHS scenarios (see Results). Because critical habitat alone cannot ameliorate all non-habitat based stressors to spotted owl populations, it was necessary to establish barred owl encounter rates that we believed were both plausible, and could, along with the critical habitat designation, lead to recovery of the spotted owl.

We made modeling region-specific decisions about barred owl encounter probabilities based on barred owl encounter probabilities estimated from studies within each modeling region [38] and the HexSim results from Stage1 (see below). We established a maximum encounter probability of 0.375 because population performance ranged from marginal to poor at higher barred owl encounter probabilities (see below). For some modeling regions with currently-estimated barred owl encounter probabilities greater than 0.375, this resulted in a substantial reduction in the barred owl encounter probabilities through time. Furthermore, we believed that achieving these barred owl encounter probabilities was plausible. For modeling regions with currently-estimated barred owl encounter rates less than 0.375, we generally assumed that barred owl encounter probabilities would remain similar to those currently estimated or would increase slightly over time and could potentially be maintained at those levels through management actions (see Table 5). In HexSim simulations, estimated current modeling region-specific barred owl encounter probabilities were inserted at time step 40 and the final probabilities were inserted at time step 60.

For Stages2 and 3, environmental stochasticity was added to the HexSim model by allowing survival rates to vary by up to 2.5% per year, and fecundity to vary by 50% per year. Stochastic survival and reproductive rates were selected independently (e.g., a good year for survival did not imply a good year for reproduction) (S1 Appendix).

Adding stochasticity increases variability within and among HexSim replicates. To adequately assess these more variable results, we ran 100 replicates of each candidate critical habitat network by two habitat change scenarios. Each replicate was run for 350 time steps. Initial evaluations of 100 replicates showed that the grand population mean was relatively stable with 100 replicates and 350 time steps.

We evaluated the following range-wide population performance metrics for Stages2 and 3: 1) total (mean of 100 replicates) population size at time step 350; 2) percent population change between time step 50 and time step 350; 3) percentage of simulations during which the range-wide population fell below 1,250 individuals; 4) percentage of simulations during which the range-wide population fell below 1,000 individuals; 5) percentage of simulations during which the range-wide population fell below 750 individuals; and 6) the grand mean of the population between time steps 150 and 350. Except for the second metric (percent change between time steps 50 and 350) all other metrics were derived from time steps 150 through 350. In most cases, HexSim simulations achieved steady-state by time step 150, and thus all but one of these metrics could be used to quantify the relative steady-state population size and distribution associated with a network.

The threshold population sizes of 1,250, 1,000, and 750 represented population sizes that represented overall population risk thresholds. Connectivity/isolation, demographic stochasticity, competition, and other factors are more likely to have deleterious impacts on small populations. Furthermore, such population sizes would likely result in large areas of the currently-occupied range becoming unoccupied by owls. Although arbitrary, these thresholds provide a consistent way to compare the relative risk of various networks and scenarios.

For each modeling region we evaluated the following population performance metrics for Stages2 and 3: 1) percentage of replicates during which the population fell below 250 individuals; 2) percentage of replicates during which the population fell below 100 individuals; 3) percentage of replicates that went to extinction; 4) mean (of the 100 replicates) population size at time step 350; and 5) grand mean of population size from time steps 150 to 350. We interpreted the percentage of simulations during which the population fell below each of the threshold modeling region population sizes to be equivalent to the probability of moderate population risk (250 females), high population risk (100 females) and extinction risk (0 females). We used these probability of population risk and extinction risk metrics to compare population results among networks; however, (unlike range-wide comparisons) we were unable to establish limits or *a priori* criteria for comparing modeling region-specific results because of the high variability in extent (area) and population sizes among modeling regions. Instead, we used the differences between risk probabilities to compare results among networks within modeling regions.

We used a combination of quantitative output from HexSim and professional judgment to evaluate composite networks and the NWFP by RHS and barred owl scenarios. We considered classifying HexSim output into categories representing the degree to which recovery goals were likely to be met. However, we did not carry through with this because there were circumstances when two results differed markedly, but both might be categorized as high risk (e.g., 33% vs. 78% of replicates falling below 250 individual females in a modeling region). In cases like this, we held that 33% was much less risk than 78%. Therefore, we evaluated both the raw output data for each metric, as well as ranking each of the reserve scenarios. The rankings provided a relatively simple and consistent method to evaluate the performance of each scenario. We also estimated the difference in population performance between optimistic and pessimistic scenarios within each candidate critical habitat network and ranked the absolute value of the differences. This was done to evaluate how reliant a network’s performance was on a particular RHS scenario–or its potential vulnerability to future uncertainty in RHS change. That is, if, within a network, population performance metrics were relatively similar (less variable) and relatively good in both optimistic and pessimistic RHS scenarios, it would suggest that that network was more resilient to uncertainty in future habitat conditions.

## Results

### Relative habitat suitability models

Because the primary objective of the RHS modeling was to provide accurate prediction of RHS, we focused primarily on the evaluation of model performance and the distribution of RHS range-wide and among modeling regions, rather than on describing spotted owl-habitat associations. In nine of the modeling regions the amount of NR habitat or a related covariate (NR edge) was the most influential covariate in the model, and it was the second and third most influential in the other two modeling regions (S1 Appendix). Of the abiotic covariates, slope position was had the largest influence in nine modeling regions (S1 Appendix).

**Fig 6:**
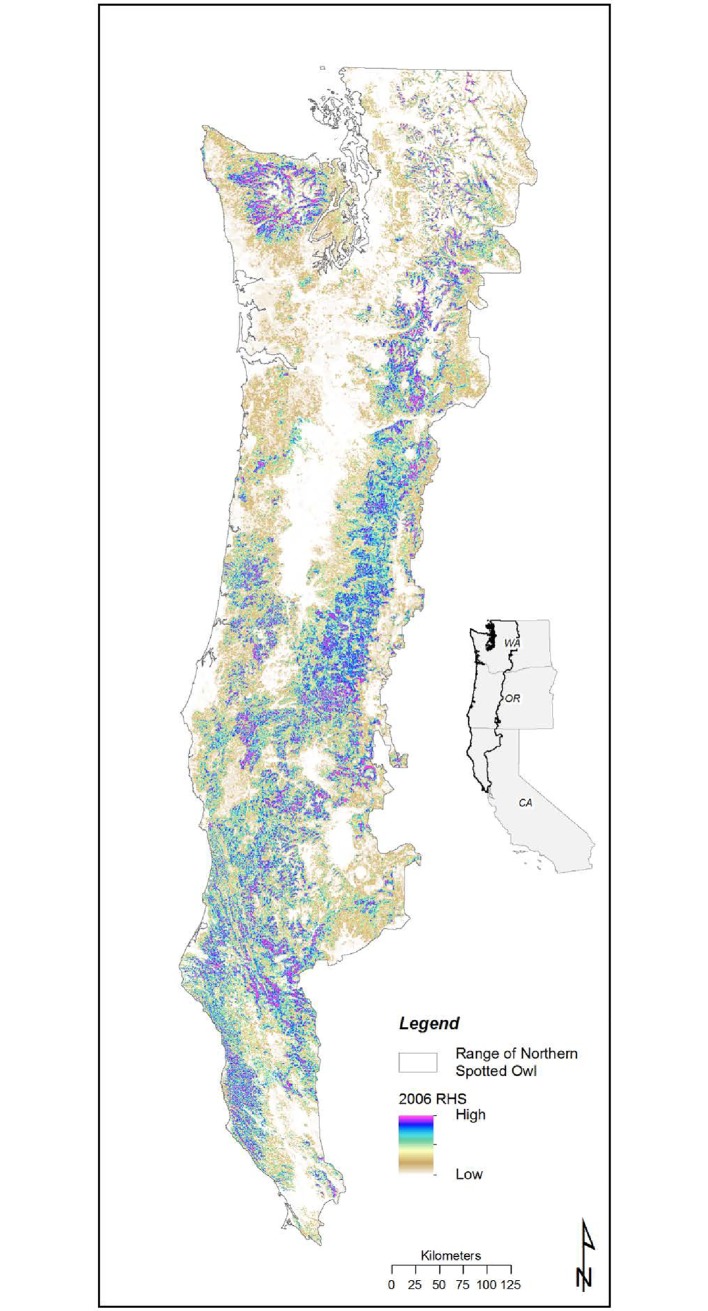
Map of relative habitat suitability throughout the geographic range of the northern spotted owl.

Overall, RHS models had good-to-excellent discrimination ability, good generality, and were well-calibrated. AUC values of full models (all data) varied between 0.76 for the RDC region and 0.93 for the WCN (Table 6). AUC values were highly correlated with the percentage of each modeling region comprised of RHS values >30, >40, and >50 (r^2^ = 0.9685, 0.9649, 0.9574, respectively). Hence, variation in AUC values among modeling regions appeared to have less to do with model discrimination ability (i.e., the quality of the model) and more to do with the quantity of suitable habitat in each modeling region. See ([48], Appendix C) for specific variables included in each region’s model. Washington had much less high RHS value area than did either Oregon or California (Fig 6).

**Table 6:** Relative habitat suitability model evaluation statistics among 11 modeling regions.

Modeling Region	AUC	Gain
**ECN**	0.879	0.842
**ECS**	0.889	0.954
**ICC**	0.820	0.543
**KLE**	0.830	0.605
**KLW**	0.769	0.396
**NCO**	0.899	1.057
**ORC**	0.863	0.810
**RDC**	0.760	0.335
**WCC**	0.892	1.024
**WCN**	0.932	1.393
**WCS**	0.758	0.345

Area under the receiver operator curve (AUC) and gain.

**Fig 7:**
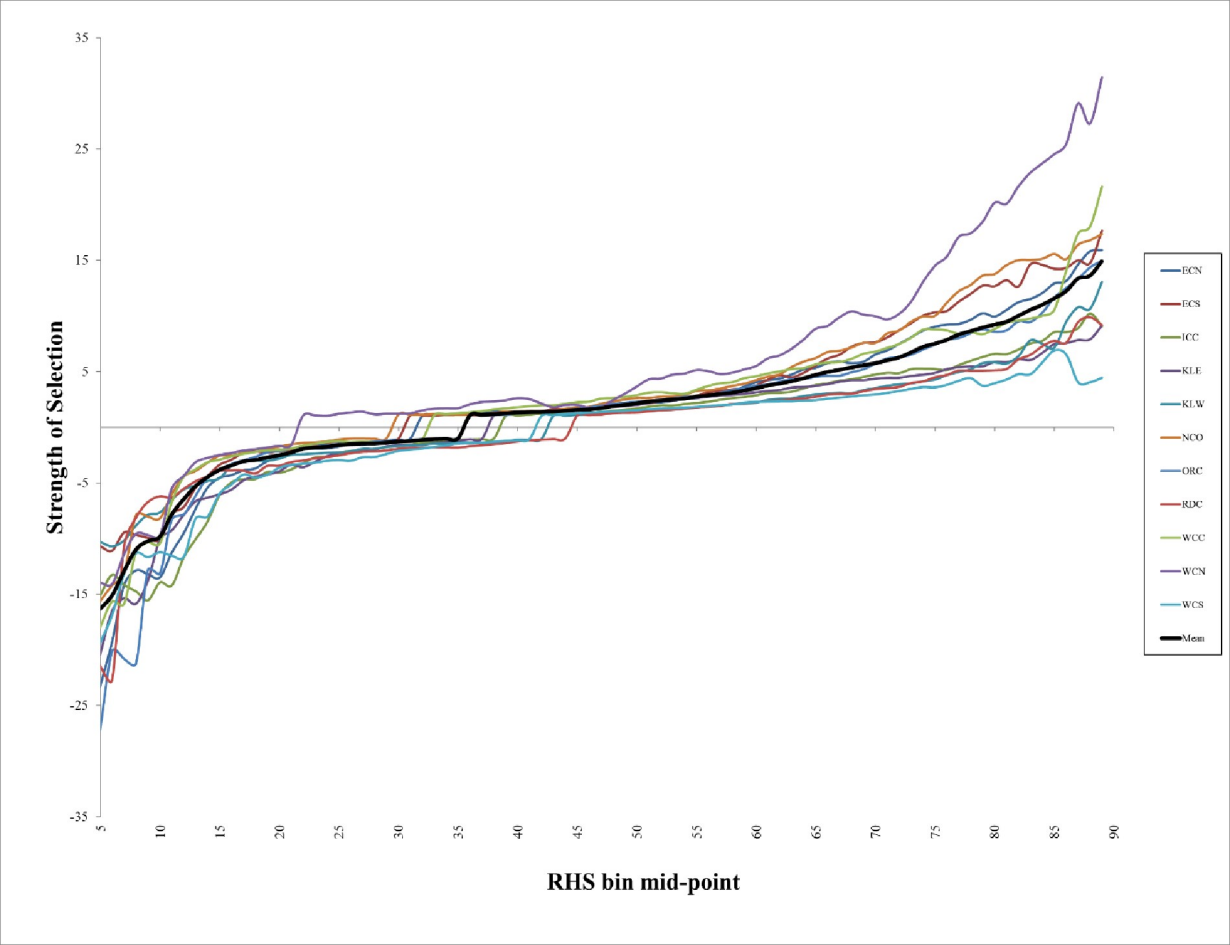
Northern Spotted Owl strength of selection (SOS) by relative habitat suitability (RHS) bin mid-point and modeling region. SOS is estimated by dividing the proportion of northern spotted owl site centers within a RHS bin by the proportion of the modeling region comprised of that bin (and thus represent relative densities of site centers). For values <1, we divided the SOS value into -1 to allow values <1 and >1 the potential to vary to the same extent. Without doing this, values <1 are constrained to be between 0 and 1, whereas values >1 can grow nearly infinitely. Legend acronyms are the 11 modeling regions and the mean of all regions combined.

Strength of selection (SOS) analyses showed that models from all regions were well-calibrated, with the relative density of owl site centers being much greater than expected in areas with RHS >60 (mean SOS for all modeling regions at RHS of 60 = 3.5 (SE = 0.32)) and much lower than expected in areas with RHS < 0.2 (mean SOS for all modeling regions at RHS of 20 = -2.77 (SE = 0.27; Fig 7)). Among modeling regions there was little variation in the magnitude of differences in relative density of owl site centers in low RHS areas, high RHS areas, and the point at which site center relative densities were approximately proportion to the extent of the RHS bin (i.e., where SOS = 1). Mean RHS where modeling region SOS values crossed from being slightly lower than expected relative densities to slightly higher than expected relative densities was between 35 and 36. SOS curves were similar among the 11 modeling regions (Fig 7).

Cross-validation revealed only very small differences in the percentages of spotted owl site centers classified among 10 equal-sized RHS bins between the full model (using all of the spotted owl locations) and the cross-validated (CV) models (S1 Appendix). The maximum percentage point difference (percentage of observations from the full model minus percentage of observations CV model) was 11.1, and the mean difference of the absolute values among modeling regions ranged from 1.6 (for the KLW) to 4.5 (for the WCN; (S1 Appendix)).

We obtained 916 independent spotted owl site center locations from the ORC, KLE, KLW, and RDC modeling regions. The percentage of spotted owl sites in each of five RHS bins for the training data and test data were very similar for all comparisons (Table 7), with 4 of 20 differences exceeding four percentage points and 11 of 20 differences being less than two percentage points.

**Table 7:** Comparison of percentage of northern spotted owl site center locations from 1996 training sites versus independent test sites from 2006 among five relative habitat suitability (RHS) bins for four modeling regions.

	Oregon Coast		Western Klamath		Eastern Klamath		Redwood Coast		Range-wide	
	Train	Test	Train	Test	Train	Test	Train	Test	Train	Test
**RHS bin**	247	169	358	136	375	108	392	284	2742	916
**0–20**	7.3	7.1	8.7	2.2	6.1	4.6	4.8	3.2	6.1	4.6
**20–40**	19.0	23.1	18.2	19.8	14.1	20.4	13.8	12.7	16.5	17.8
**40–60**	35.6	35.5	38.5	46.3	38.4	39.8	42.1	44.7	36.7	41.8
**60–80**	32.8	30.2	33.5	30.8	38.7	35.2	37.2	37.7	36.7	33.8
**80–100**	5.3	4.1	1.1	0.74	2.7	0	2.0	1.8	4.0	1.2

Numbers immediately below Train and Test are sample sizes of Training and independent Test nests.

More than 64% of the geographic range of the spotted owl had RHS values <20, whereas 31% of the area had RHS values between 20 and 60, and 5.2% had RHS values >60 (Table J in S1 Appendix). Nonetheless, there was wide variation in the distribution of RHS among modeling regions (Table J in S1 Appendix). The WCN, NCO, ECS, WCC, and ECN modeling regions had more than 70% of their area with RHS values <20, but the KLW and RDC had 36.99% and 43.36% of their area with RHS values <20, respectively. The percentage of modeling regions with RHS values >60 ranged from 2.83 (WCN) to 8.04 (RDC) (Table J in S1 Appendix).

#### Network sizes and relative habitat suitability

The Zonation networks we evaluated ranged from 5.57 to 11.24 million ha when Congressionally Reserved lands were included (Table 8). Because Congressional Reserves are existing protected areas and will continue to provide some habitat value for the spotted owl in the future similar to what they currently provide (major deviations from climate change-induced increases in canopy fires notwithstanding), when we henceforth reference network sizes and performance, they always include Congressional Reserved lands, even when they were not selected for inclusion in a critical habitat composite. Zonation ALL and PUB networks differed by less than 1% in size for Z30 and Z50, but Z70PUB was 6.5% larger than Z70ALL (Table 8). The percentage of 1996 spotted owl sites that were included in the NWFP was 46%, and varied from 50% to 88% in Zonation networks (Table 8).

**Table 8:** Comparison of 6 candidate critical habitat networks (we developed) by size and percent of 1996 spotted owl sites used in model development that occur within the network.

Network	Network scenario size (million hectares)	Percent of 1996 spotted owl sites
NWFP	6.63	46
1992 Critical Habitat	5.75	44
2008 Critical Habitat	5.17	37
Z30 All lands	5.61	50
Z50 All lands	7.80	71
Z70 All lands	10.55	87
Z30 Public lands	5.57	51
Z50 Public lands	7.82	73
Z70 Public lands	11.24	88

We also include the Northwest Forest Plan (NWFP), and critical habitat designated in 1992 and 2008 for comparison. Z = Zonation-derived networks. The number after Z represents the percentage of habitat value and ALL = no prioritization of lands included, whereas PUB = public lands were prioritized and non-public lands were only included if the goal could not be met with public lands.

Reserve networks of vastly different sizes could potentially function very similarly if the smaller of the two was primarily comprised of high RHS and the larger had much more low RHS. Therefore, we evaluated both network size and amount of various RHS classes contained within the network. Zonation networks included a higher percentage of high RHS than the NWFP and ALL networks included more high RHS than PUB networks (Table K in S1 Appendix).

Approximately 1.21 million ha, or 5.2% of the total area, within the more than 23 million-ha range of the spotted owl had RHS values ≥60, about 58,890 ha had RHS between 80 and 90, and only about 2,300 ha had RHS ≥90. Approximately 14.9 million ha, or 64.2% of the total area, had RHS <20 and about 12 million ha had RHS <10. All composite networks contained >70% of the areas with RHS ≥60 (Table K in S1 Appendix). Composite 1, the largest composite network, contained the most area of the highest RHS categories (range = 86–98% for the four bins with RHS >60). Composite 8 was the smallest composite network and contained the least area of the highest RHS categories (range = 72–81%). There was relatively little variation in the percentage of RHS 0–9.99 included in composite networks (range = 18–22%). There were, however, relatively large differences in the percentage of RHS between 10–19.99 and 20–29.99 among composite networks (ranges = 26–41% and 35–51%, respectively). As Zonation-only networks got larger, and when comparing PUB to ALL networks including the same amount of habitat value, most of the differences were in the percentage of the lowest RHS category (0–9.99) included. For example Z70ALL included 8% of the 0–9.99 RHS area whereas Z70PUB included 25% of that RHS category.

#### Spatially explicit individual-based population model

The baseline HexSim simulations without environmental stochasticity, in which barred owl impacts were introduced at time step 40, then held static at 2008-estimated rates, produced an estimated total female spotted owl population size of 675 within the eight DSAs for which empirical abundance estimates were available. This estimate was 89% of the empirical population estimate from those DSAs (S1 Fig). Similarly, the distribution of dispersal distances of simulated owls was quite similar to those estimated empirically ([98]; S2 Fig). Sensitivity analyses at the modeling region and range-wide scales revealed appreciable effects of varying parameter values for reproduction (Table K, columns 7A-B, in S1 Appendix) and survival or parameters that influenced survival (Table K, columns 3A-D; 4A-C; 6A-B in S1 Appendix). Nonetheless, varying survival, and/or parameters that influenced survival had the largest impacts (Table K in S1 Appendix).

#### Stage1 simulation results

Stage1 simulations revealed a strong influence of barred owl encounter rates, relative to RHS change and network size, on estimated spotted owl population performance. In general, scenarios with the largest barred owl encounter rate (STVA4) resulted in the smallest (range = 87–94% reduction in population size among 21 such STVA4 by HAB scenarios) and least variable estimated spotted owl population sizes, regardless of network or RHS scenario (Table 9). In scenarios with intermediate barred owl encounter rates (STVA2 and STVA3), estimated spotted owl population sizes were much more variable among networks and RHS scenarios–with larger networks and habitat scenarios that maintained more high-RHS area (HAB2 and HAB3) having larger population sizes than smaller networks (Table 9). For example, simulated populations were estimated to decline by between 16% (Z70ALL) and 54% (NWFP) for the HAB1 scenario when barred owl encounter probabilities were 0.25 (STVA3). When barred owl encounter probabilities were maintained at their currently-estimated levels (STVA3) and with HAB1, simulated spotted owl populations were estimated to decline by 40% (Z70ALL and Z70PUB) to 66% (NWFP). Within a network, such as the Z30ALL, and with the same RHS scenario (HAB1 in this case), spotted owl population declined an estimated 30% to 91% depending on the barred owl scenario (0.0 and 0.5 encounter rates in this example, respectively). Likewise, under the same HAB1 RHS scenario and with barred owl encounter rates held at 0.25 in all regions (STVA3), spotted owl populations declined an estimated 16% to 54% with the smallest population declines in the largest networks (Table 9).

**Table 9:** Phase 1 HexSim modeling results showing the percentage of the time-step 50 range-wide population size that was realized at time-step 250 (mean of 5 replicates) among 7 candidate critical habitat networks, various barred owl encounter rates, and relative habitat suitability (RHS) change scenarios.

		Network						
Barred Owl Encounter Probability	RHS Scenario	NWFP	Z30all	Z50all	Z70all	Z30pub	Z50pub	Z70pub
STVA1 (0.0)	HAB1	56.7	70.0	90.0	102.3	70.5	87.9	98.2
STVA2 (Current)	HAB1	33.7	45.4	55.2	60.2	40.0	50.8	60.0
STVA3 (0.25)	HAB1	46.4	58.4	74.7	83.7	58.8	70.1	76.9
STVA4 (0.5)	HAB1	5.8	8.8	10.3	12.7	7.9	9.1	11.5
STVA1 (0.0)	HAB2	81.3	86.3	94.7	104.1	84.4	89.9	97.4
STVA2 (Current)	HAB2	47.3	55.0	60.4	59.4	50.6	51.9	58.5
STVA3 (0.25)	HAB2	64.2	69.9	77.0	84.0	65.7	73.5	77.4
STVA4 (0.5)	HAB2	9.0	10.5	9.5	13.2	8.7	8.8	9.3
STVA1 (0.0)	HAB3	95.3	94.7	102.2	103.3	96.8	102.0	100.9
STVA2 (Current)	HAB3	59.8	56.3	58.7	60.5	58.0	57.7	63.0
STVA3 (0.25)	HAB3	79.6	72.3	77.0	81.5	74.7	76.5	80.6
STVA4 (0.5)	HAB3	10.7	11.8	13.2	11.8	12.2	12.1	10.3

HAB1 = scenario in which all non-network lands with RHS values >35 were reduced such that they had values of 34.9, otherwise RHS remained constant. HAB2 = scenario in which all non-network public lands with RHS >50 were maintained, otherwise non-network lands with RHS >35 were reduced to 34.9; all other lands remained the same. HAB3 = scenario in which all non-network lands with RHS >50 were maintained, otherwise non-network lands with RHS >35 were reduced to 34.9; all other lands remained the same. For these results HAB3 was the relative habitat suitability change that was used (i.e., no change was made to lands within networks and all lands with RHS ≥ 50 were maintained. Lands not in networks that had RHS values from 35–49.9 were truncated to 34.99, otherwise RHS did not vary.

In general, among similar STVA (except STVA4 which had little variation in results) and RHS scenarios, simulated owls performed worse in the NWFP than the Zonation networks, and best in the largest networks (Z70ALL and Z70PUB). If estimated current barred owl encounter rates were to be maintained (STVA2) and under HAB1, the NWFP network was estimated to have spotted owl populations decline by 66%, whereas owls in the Z70ALL network declined by 40%. Lastly, without barred owls on the landscape (STVA1), and under the same RHS change scenario, spotted owl populations under the NWFP network were estimated to decline by 43%, but increase by 2% under the Z70ALL network (Table 9).

The population stability metrics (first year that lambda became stable and number of years it was stable) were relatively uninformative because they were very similar for most RHS by STVA by network combinations. Therefore, we put little emphasis on those metrics.

For individual modeling regions, Stage1 modeling suggested that spotted owls in the ICC, KLE, KLW, and WCS modeling regions were the most stable and least prone to fall below either quasi-extinction threshold (250 or 100 individuals) except under STVA4. Under STVA4, 76 of 77 modeling region by network evaluations fell below 250 individuals during all five replicates, and the 77^th^ did so during four of five replicates. The RDC modeling region also generally had a low quasi-extinction (100 and 250) rate, but under the NWFP network, populations frequently fell below those thresholds. In contrast, the WCC, WCN, and ECS modeling regions most frequently (60–100% of replicates among networks) fell below quasi-extinction thresholds (especially 250), even under HAB3 and STVA1 (no barred owls). Similar to the range-wide results, STVA4 scenarios resulted in populations falling below quasi-extinction 250 in 100% of replicates for 221 of 231 modeling region by network by HAB scenarios. Among the various networks, and except for STVA4, owl population metrics among modeling regions generally performed best in the larger networks and more poorly in smaller networks.

#### Stages 2 and 3

After evaluating Stage1 modeling-region-specific results, we developed 11 additional composite scenarios. Composites 1 and 2 represented lower and higher estimated risk to spotted owls, whereas Composites 3–11 represented various refinements to Composites 1 and 2 or suggestions from federal land management agencies. Because Stage1 results revealed that under STVA4 barred owl impacts overwhelmed variation in RHS change and network size and spacing we made modeling region-specific decisions on the barred owl encounter rates to carry through to Stages2 and 3, including an upper limit of 0.375 for any modeling region. We decreased barred owl encounter probabilities in 3 of 11 modeling regions and increased encounter probabilities in 8 modeling regions (Table 10). Decreases ranged from 0.335 (0.71 to 0.375 in the OCR) to 0.065 (0.315 to 0.25 in the KLW). Increases ranged from 0.005 (0.245 to 0.25 in KLE) to 0.079 (0.296 to 0.375 in ECN). Mean absolute value of change among modeling regions was 0.081 (SD = 0.091).

**Table 10:** Barred owl (STVA) encounter rates by modeling region.

Modeling Region	Estimated STVA	HexSim STVA
OCR	0.71	0.375
KLW	0.315	0.25
RDC	0.205	0.25
KLE	0.245	0.25
ICC	0.213	0.25
WCS	0.364	0.375
WCC	0.32	0.375
WCN	0.32	0.375
NCO	0.505	0.375
ECN	0.296	0.375
ECS	0.18	0.25

Estimated STVA are values estimated from Forsman et al. (2011), and HexSim STVA are the values used for Phase 2 and 3 HexSim modeling.

#### Modeling region comparisons

Under the optimistic RHS scenario, simulated owl populations in the WCN, WCC, and NCO modeling regions went to extinction in more than 75% (75–82%), 13% (13–27%), and 11% (11–22%) of replicates under all networks (Table 11), respectively. In all other modeling region by network comparisons, no more than 2% of replicates resulted in simulated populations going to extinction, with the vast majority never going to extinction. However, in the ECN, ECS, NCO, ORC, WCC, and WCN modeling regions, >99% of simulated populations fell below 250 individuals in all networks evaluated. In the WCS from 44% (Composite 3) to 52% (Composite 8) of simulations had estimated populations falling below 250. In the ICC, KLE, KLW, and RDC modeling regions, from 0 to 18% of simulations fell below 250 among networks (Table 11).

**Table 11:** Estimated northern spotted owl population responses, in modeling regions, among candidate critical habitat networks for the *optimistic* relative habitat suitability change scenarios.

	Network											
	NWFP	Composite 1	Composite 2	Composite 3	Composite 4	Composite 5	Composite 6	Composite 7	Composite 8	Composite 9	Composite 10	Composite 11
**# simulations with N<250**												
ECN	100	100	100	100	100	100	100	100	100	100	100	100
ECS	100	100	100	100	100	100	100	100	100	100	100	100
ICC	1	3	3	1	3	5	4	5	6	1	3	0
KLE	5	8	8	13	12	16	17	13	18	14	9	6
KLW	4	6	3	8	8	8	11	6	11	8	5	3
NCO	100	100	100	100	100	100	100	100	100	100	100	100
ORC	100	99	99	100	100	99	98	100	100	100	99	99
RDC	12	7	11	6	13	11	10	9	11	8	3	3
WCC	100	100	100	100	100	100	100	100	100	100	100	100
WCN	100	100	100	100	100	100	100	100	100	100	100	100
WCS	46	45	45	44	47	51	48	45	52	50	42	45
**# simulations with N<100**												
ECN	96	86	92	90	95	90	85	92	92	92	96	90
ECS	97	79	85	85	81	84	80	87	87	84	89	84
ICC	0	1	0	0	0	0	0	0	0	1	0	0
KLE	0	1	0	1	0	0	1	2	0	0	1	0
KLW	0	1	0	0	0	0	1	1	0	0	1	0
NCO	99	99	100	99	100	100	99	100	100	100	100	100
ORC	69	61	56	61	65	64	61	65	67	63	61	60
RDC	0	1	0	0	0	1	0	0	0	1	0	0
WCC	100	100	100	100	100	100	100	100	100	100	100	100
WCN	100	100	100	100	100	100	100	100	100	100	100	100
WCS	9	10	8	9	11	12	11	12	16	6	4	4
**# Extinctions**												
ECN	0	2	0	1	2	1	2	2	1	0	1	1
ECS	0	0	0	0	0	0	0	0	0	0	0	0
ICC	0	0	0	0	0	0	0	0	0	0	0	0
KLE	0	0	0	0	0	0	0	0	0	0	0	0
KLW	0	0	0	0	0	0	0	0	0	0	0	0
NCO	22	18	16	19	20	22	15	15	17	17	11	15
ORC	0	1	0	0	0	0	1	0	0	0	0	0
RDC	0	0	0	0	0	0	0	0	0	0	0	0
WCC	17	19	19	16	26	27	22	21	25	13	18	15
WCN	81	75	79	78	78	82	76	73	80	77	79	82
WCS	0	0	0	0	0	0	0	0	0	0	0	0
**N**_**350**_												
ECN	78 (68–88)	98 (87–110)	89 (79–99)	89 (78–100)	83 (72–93)	85 (73–96)	84 (72–96)	94 (81–107)	77 (67–86)	91 (79–102)	84 (74–94)	90 (79–101)
ECS	134 (126–142)	149 (140–158)	141 (132–149)	140 (132–148)	138 (130–146)	135 (125–145)	135 (127–143)	139 (130–148)	126 (116–135)	135 (127–143)	140 (131–148)	145 (135–155)
ICC	921 (869–972)	970 (917–1022)	915 (862–968)	942 (891–993)	920 (873–967)	909 (848–969)	906 (854–958)	921 (865–978)	831 (773–889)	895 (843–948)	906 (850–963)	963 (904–1023)
KLE	705 (658–751)	749 (702–796)	714 (670–758)	717 (670–764)	699 (654–743)	674 (624–724)	702 (653–751)	690 (637–744)	647 (596–699)	685 (637–733)	695 (647–743)	747 (695–800)
KLW	861 (804–919)	882 (828–936)	844 (790–898)	840 (787–892)	836 (784–888)	833 (773–893)	825 (766–883)	829 (769–890)	771 (712–831)	819 (762–877)	822 (765–879)	881 (819–942)
NCO	49 (39–60)	46 (37–56)	56 (46–66)	52 (41–63)	46 (37–56)	50 (40–61)	48 (40–57)	48 (38–58)	47 (37–56)	48 (38–57)	47 (39–55)	54 (44–64)
ORC	167 (145–190)	176 (152–201)	180 (158–201)	173 (151–196)	156 (136–176)	161 (136–186)	165 (140–189)	170 (146–193)	146 (127–165)	179 (156–202)	175 (152–198)	190 (165–215)
RDC	673 (633–713)	750 (707–794)	664 (624–703)	722 (681–764)	714 (675–754)	704 (657–751)	719 (674–764)	747 (701–793)	686 (638–733)	720 (673–768)	742 (696–789)	754 (704–804)
WCC	27 (22–32)	31 (26–36)	31 (25–36)	31 (26–36)	22 (17–27)	26 (20–31)	26 (20–31)	30 (24–36)	22 (18–26)	30 (25–36)	29 (24–34)	30 (24–35)
WCN	4 (2–5)	4 (3–6)	4 (2–5)	5 (3–6)	4 (3–5)	4 (3–6)	4 (3–5)	5 (3–7)	4 (2–5)	4 (3–6)	5 (3–6)	4 (3–6)
WCS	475 (418–531)	478 (421–534)	478 (425–530)	493 (431–555)	437 (388–486)	460 (399–520)	454 (397–510)	475 (418–533)	417 (368–466)	492 (430–554)	460 (409–511)	517 (450–584)

NWFP refers to the Northwest Forest Plan. N_350_ = mean (95% CI) population size at time-step 350 among 100 replicate simulations.

For the optimistic RHS scenario, estimated population size at time step 350 was generally smallest in modeling regions under Composite 8 and NWFP, and largest under Composites 1 and 11 (Table 11). As with the range-wide population performance metrics, there was relatively little variation in population performance among networks under the optimistic RHS scenario (Table 11).

Under the pessimistic RHS scenario, simulated spotted owl populations in the ECS, ICC, KLE, KLW, RDC, and WCS never went to extinction among the 100 replicates in any of the networks (Table 12). Simulated owl populations in the WCN were most prone to extinction, ranging from 75% (Composite 4) to 84% (Composites 8 and 9) of simulations. However, in every network and modeling region, from 3 to 100% of simulations fell below 250 individuals, with the KLW and ICC populations falling below 250 individuals the fewest times (Table 12), and the ECN, ECS, NCO, ORC, WCC, and WCN populations falling below 250 individuals during ≥98% of replicates in all networks. Mean estimated population size at time step 350 within modeling regions was generally largest under Composites 1, 4, 10, and 11 (Table 12).

**Table 12:** Estimated northern spotted owl population responses, in modeling regions, to potential critical habitat networks for the *pessimistic* relative habitat suitability change scenarios.

	Network											
	NWFP	Composite 1	Composite 2	Composite 3	Composite 4	Composite 5	Composite 6	Composite 7	Composite 8	Composite 9	Composite 10	Composite 11
**# simulations with N<250**												
ECN	100	100	100	100	100	100	100	100	100	100	100	100
ECS	100	100	100	100	100	100	100	100	100	100	100	100
ICC	44	14	21	7	15	16	19	16	26	20	17	17
KLE	87	34	51	26	30	50	43	39	41	36	36	32
KLW	22	5	10	3	7	10	6	9	15	11	9	6
NCO	100	100	100	100	100	100	100	100	100	100	100	100
ORC	100	98	99	100	99	100	100	100	99	100	100	98
RDC	92	44	87	50	46	45	48	43	47	48	45	59
WCC	100	100	100	100	100	100	100	100	100	100	100	100
WCN	100	100	100	100	100	100	100	100	100	100	100	100
WCS	73	54	74	54	55	60	64	54	65	61	58	59
**# simulations with N<100**												
ECN	100	87	94	100	94	99	97	96	100	98	100	100
ECS	100	96	98	99	98	100	99	98	100	100	99	97
ICC	0	0	0	0	1	1	2	0	1	0	0	1
KLE	6	2	1	0	2	5	1	2	3	1	1	0
KLW	0	0	0	0	1	0	0	0	0	0	0	1
NCO	100	97	100	100	99	100	99	100	100	100	100	100
ORC	70	48	65	65	56	72	69	58	59	65	59	56
RDC	6	2	5	0	2	2	4	1	1	1	2	2
WCC	100	100	100	100	100	100	100	100	100	100	100	100
WCN	100	100	100	100	100	100	100	100	100	100	100	100
WCS	16	15	23	9	16	16	18	18	17	17	13	10
**# Extinctions**												
ECN	3	2	1	3	2	5	1	2	0	2	2	2
ECS	0	0	0	0	0	0	0	0	0	0	0	0
ICC	0	0	0	0	0	0	0	0	0	0	0	0
KLE	0	0	0	0	0	0	0	0	0	0	0	0
KLW	0	0	0	0	0	0	0	0	0	0	0	0
NCO	19	9	21	6	18	20	18	26	21	29	23	18
ORC	0	0	0	0	1	1	0	0	0	0	0	0
RDC	0	0	0	0	0	0	0	0	0	0	0	0
WCC	26	25	26	30	32	29	27	22	45	27	19	29
WCN	78	76	77	80	75	79	81	77	84	84	78	81
WCS	0	0	0	0	0	0	0	0	0	0	0	0
**N**_**350**_												
ECN	43 (37–49)	89 (78–100)	67 (58–76)	44 (38–51)	68 (58–77)	49 (43–56)	63 (54–73)	69 (60–78)	57 (49–65)	60 (52–68)	64 (56–72)	64 (56–73)
ECS	74 (69–80)	122 (114–129)	106 (99–113)	115 (107–123)	122 (112–131)	102 (95–110)	114 (106–122)	115 (107–123)	107 (101–114)	109 (103–116)	120 (112–128)	120 (112–128)
ICC	456 (430–482)	640 (604–677)	520 (489–551)	626 (589–663)	632 (587–678)	659 (617–701)	611 (567–654)	613 (572–655)	516 (481–550)	584 (552–616)	616 (576–656)	608 (568–647)
KLE	341 (316–366)	538 (500–575)	421 (392–451)	512 (477–547)	527 (486–569)	442 (408–477)	492 (452–533)	491 (454–528)	444 (412–476)	490 (457–523)	533 (492–574)	549 (508–591)
KLW	616 (575–657)	814 (764–864)	629 (587–671)	771 (723–818)	785 (727–843)	791 (735–847)	766 (709–823)	757 (706–808)	645 (599–691)	721 (677–765)	784 (725–844)	815 (755–875)
NCO	48 (39–57)	61 (49–72)	39 (31–47)	50 (41–59)	51 (40–61)	48 (38–57)	46 (37–55)	39 (30–49)	40 (32–49)	36 (28–44)	48 (36–59)	49 (39–59)
ORC	165 (144–186)	216 (188–244)	169 (145–194)	161 (141–181)	198 (170–226)	135 (117–153)	174 (150–197)	184 (161–206)	172 (149–194)	173 (151–195)	178 (150–205)	197 (169–224)
RDC	323 (301–345)	454 (426–483)	315 (294–337)	444 (417–472)	456 (423–488)	462 (433–491)	442 (409–475)	463 (431–496)	442 (411–473)	438 (412–464)	449 (417–481)	424 (393–454)
WCC	23 (18–27)	27 (22–32)	23 (18–28)	18 (15–22)	19 (15–23)	19 (16–22)	21 (17–26)	25 (20–30)	14 (11–18)	20 (16–24)	26 (21–31)	27 (21–32)
WCN	5 (3–6)	5 (3–6)	4 (3–6)	4 (3–5)	5 (3–7)	4 (2–5)	4 (2–5)	5 (3–6)	3 (2–4)	4 (2–5)	5 (3–6)	5 (3–7)
WCS	326 (285–367)	409 (362–455)	294 (258–330)	401 (351–451)	427 (370–484)	367 (320–414)	375 (323–427)	399 (347–450)	346 (304–388)	352 (311–393)	391 (33–443)	402 (351–453)

NWFP refers to the Northwest Forest Plan. N_350_ = mean (95% CI) population size at time-step 350 among 100 replicate simulations.

#### Range-wide comparisons

Under the optimistic RHS change scenario we found relatively small differences in owl population performance among Composites 1–11 and the NWFP (Table 13). For example, the average estimated percentage of time step 50 population size that was realized at time step 350 ranged from 54% (Composite 8) to 63% (Composite 11). Similarly, the number of replicates during which population size fell below 1,000 individuals ranged from 0 to 3. Mean population sizes at time step 350 under the optimistic RHS scenario ranged from 3,774 (Composite 8) to 4,375 (Composite 1), however, the 95% confidence intervals of all optimistic RHS scenario networks overlapped (Table 13).

**Table 13:** Estimated range-wide northern spotted owl population responses to candidate critical habitat networks for the optimistic and pessimistic relative habitat suitability change scenarios.

	NWFP	C1	C2	C3	C4	C5	C6	C7	C8	C9	C10	C11
Optimistic Scenario												
**N**_**350**_	4094	4333	4114	4204	4055	4041	4066	4149	3774	4099	4105	4375
**95% CI of N**_**350**_	3817–4371	4054–4612	3852–4377	3922–4486	3799–4312	3732–4351	3777–4356	3842–4456	3484–4065	3803–4395	3826–4384	4056–4695
**Geographic Range** **N**_**350**_**/N**_**50**_**·100**	57	62	58	60	56	57	58	61	54	60	58	63
**# Simulations with N<1250**	2	3	2	3	6	6	7	5	14	3	3	2
**# Simulations with N<1000**	1	2	0	1	1	2	2	3	2	1	1	0
**# Simulations with N<750**	0	1	0	1	0	2	1	0	0	1	0	0
**Pessimistic Scenario**												
**N**_**350**_	2420	3374	2588	3147	3289	3078	3108	3161	2787	2987	3214	3259
**95% CI of N**_**350**_	2245–2595	3141–3607	2401–2774	2927–3367	3019–3559	2850–3306	2854–3362	2922–3400	2580–2993	2788–3185	2956–3473	3000–3517
**Geographic Range** **N**_**350**_**/N**_**50**_**·100**	35	47	37	43	47	43	44	44	40	43	45	45
**# Simulations with N<1250**	40	10	21	7	16	17	17	14	18	14	15	10
**# Simulations with N<1000**	14	4	6	3	5	7	6	6	7	9	7	5
**# Simulations with N<750**	1	2	2	0	2	4	3	2	2	1	2	2

NWFP refers to the Northwest Forest Plan, and C1-C11 refer to potential critical habitat networks Composite 1 –Composite 11. N_350_ = mean population size at time-step 350 among 100 replicate simulations. N_350_/N_50_·100 = mean percentage of time-step 50’s population that was realized at time-step 350 among 100 replicate simulations.

Under the pessimistic RHS scenario, we observed more pronounced differences in owl population performance among networks (Table 13). Estimated percentage of time step 50 population size that was realized at time step 350 averaged from 35% (NWFP) to 47% (Composites 1 and 4; Table 13). Number of replicates during which the range-wide population fell below 1,000 individuals ranged from 3 (Composite 3) to 14 (NWFP). Mean population size at time step 350 under the pessimistic RHS scenario ranged from 2,420 (NWFP) to 3,374 (Composite 1); however, 95% confidence intervals overlapped for Composites 1, 3–7, and 9–11 (Composite 8’s confidence interval overlapped with all of these except for Composites 4 and 11) (Table 13).

There were no replicates in either optimistic or pessimistic RHS scenarios during which the range-wide population went to extinction. Range-wide populations fell below 750 individuals during only 29 (6 optimistic and 23 pessimistic) of 2,400 (1.21%) replicates (Table 13).

#### Efficiency

**Fig 8:**
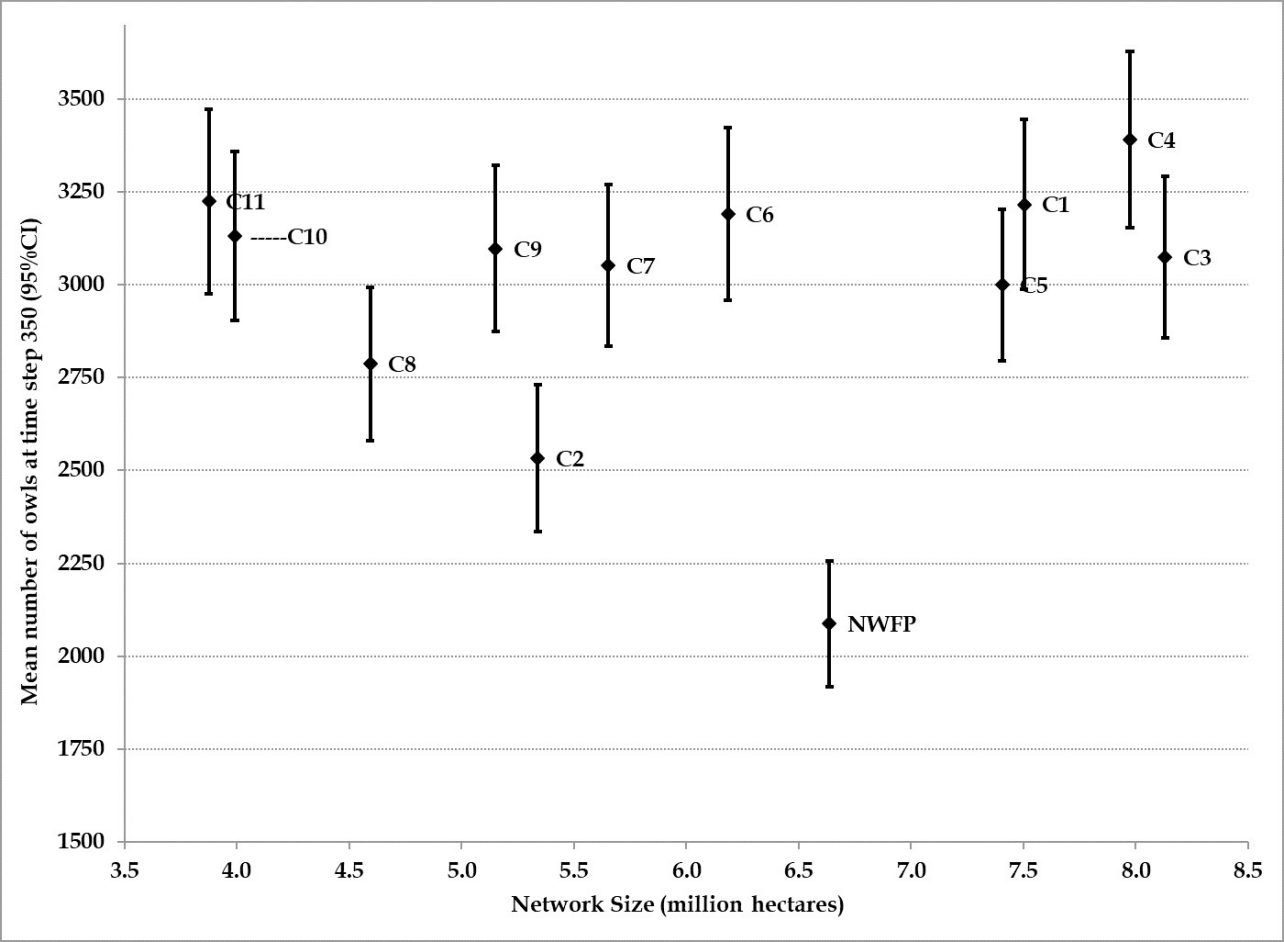
Mean and 95% confidence intervals of simulated northern spotted owl population sizes among 11 composite candidate critical habitat networks and the Northwest Forest Plan (NWFP) size, based on the pessimistic habitat change scenario.

**Fig 9:**
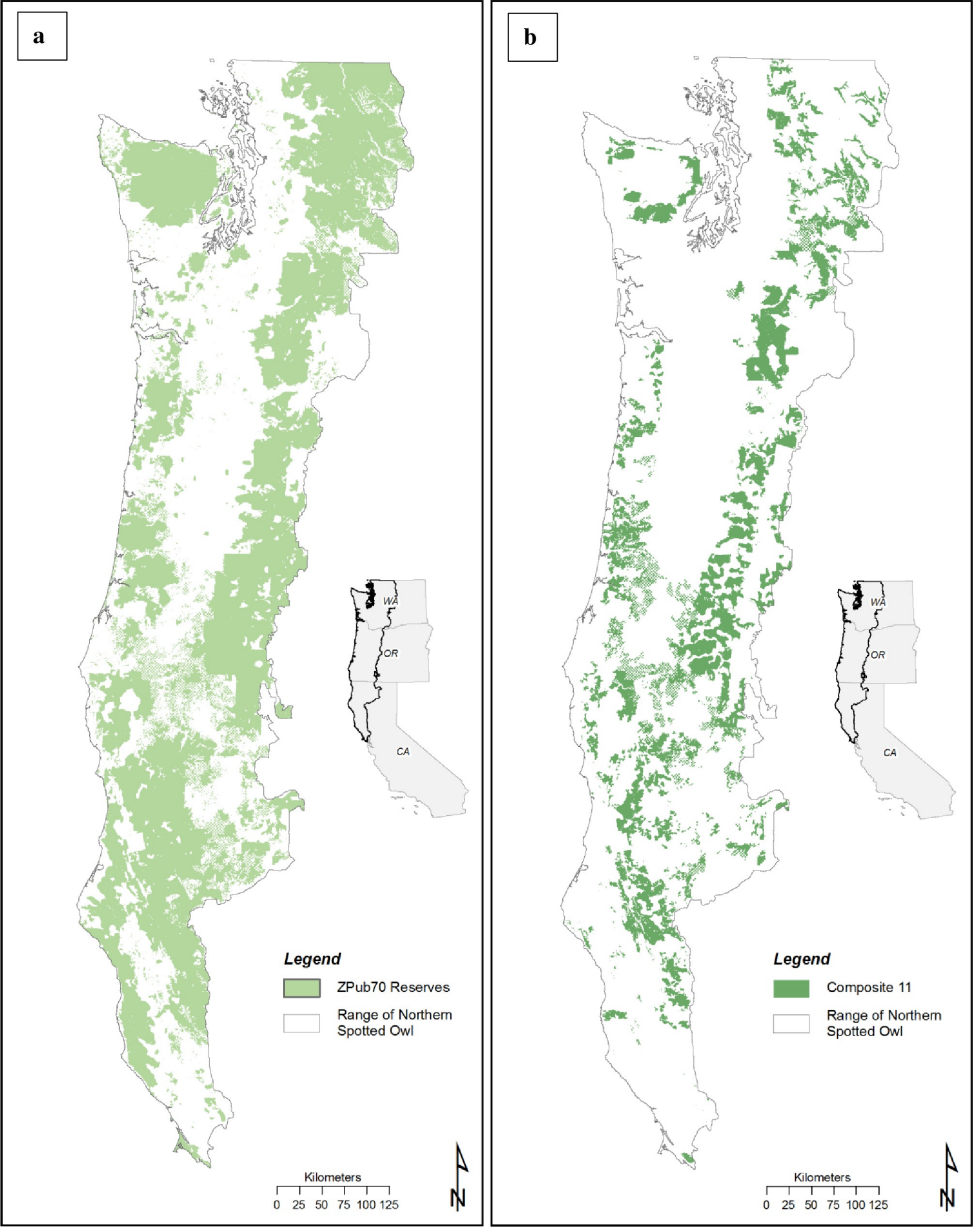
Maps of (a) Composite 11 (Critical Habitat designated in 2012), and (b) The Zonation scenario with 70% of habitat value, with emphasis on public lands. Although Z70PUB was more than twice the size of Composite 11, simulated spotted owl populations performed similarly.

Composite 11 was the most efficient of the networks we evaluated (Fig 8). Population performance metrics were nearly identical for Composite 11 and other high-performing networks, but Composite 11 was less than half the size (Fig 9) of some other high-performing networks (e.g., Composite 4).

## Discussion

The depth and breadth of research on the spotted owl range from habitat selection at different spatial scales and throughout much of the species' range (e.g., [41, 80]) to long-term demographic studies [37–40] to more recent evaluations of the impacts of competition with the congeneric barred owl [40, 50, 51]. The tremendous body of literature on spotted owls provided strong empirical underpinnings for much of our modeling. The vast majority of large-scale conservation planning projects have large data gaps, whether missing altogether, data being from locations that may not represent broader conditions in which the species lives, or from relatively short-duration studies. Thus, it is perhaps unsurprising that most such efforts rely on proxies or best guesses to fill in informational gaps or to parameterize models. We contend that, owing to the quantity and quality of data available on spotted owls, we came close to achieving the ideal of successfully combining spatial prioritization and population modeling to evaluate the likelihood of population persistence under a variety of scenarios [2, 106]. We did, however, make simplifying assumptions (e.g., no variation of barred owl encounter rates within modeling regions) when existing data could not substantiate more nuanced representations. Nonetheless, the modeling framework we created is scalable and can incorporate such data as they become available.

Our project was motivated by legal requirements under the ESA. Our modeling work was a part of this larger process and necessitated several formal legal and policy considerations. Nonetheless, our approach allowed for comparisons of candidate networks and scenarios, including those that might be considered optimistic but unrealistic from a social-politico standpoint. The ESA requires that the listing of a species, sub-species, or distinct population be made “solely on the basis of the best scientific and commercial data available” [107]. We attempted to use that same guidance in our process of developing and evaluating candidate critical habitat networks.

Our overall approach was to identify RHS throughout the spotted owl's range using MaxEnt, use that RHS map to help identify habitat networks in Zonation, and then use HexSim, an individual-based spatially-explicit population model, to evaluate spotted owl population performance among the various networks. To our knowledge, our effort is the first to use these conservation planning tools in tandem to develop and evaluate alternative networks for the designation of critical habitat. In a review of studies evaluating critical habitat in the U.S., Canada, and Australia, ours was the only one that explicitly referenced population viability [108].

Our models and approach are scalable. Thus, if and when the distribution, abundance, demographic performance, and impacts of barred owls become better understood, and in a spatially-explicit manner, we can include those more nuanced effects in HexSim. If warranted, HexSim can explicitly accommodate a two-species model [51] with barred owls using resources, breeding, dying, etc.; as opposed to our representation of them as only influencing spotted owl survival. Further, there may be alternative ways to model barred owl influence on spotted owls. For example, barred owl presence was used in HexSim to decrement spotted owl habitat quality, rather than spotted owl survival [109]. The best way to incorporate barred owls in an explicitly two-species HexSim model may yet be told from continued monitoring and from field studies on the interactions of the species. Other refinements to our modeling approach may also be possible as more is learned about spotted owls themselves. For example, a two-sex model could become possible for spotted owls, as mate finding, “divorce”, and adult dispersal become better understood.

Provided sufficient data exist, our approach to developing and evaluating candidate critical habitat networks for spotted owls could be applied to conservation and/or land use planning for other species. However, for most other species more proxies or professional opinion would likely be needed to parameterize models. Our process was repeatable and scientifically defensible and allows for evaluation of the efficacy of candidate habitat networks or reserves for meeting conservation objectives under a variety of potential future scenarios. For other species and circumstances, potential future scenarios could include urban development, energy development, habitat conversion, water diversion, livestock grazing, mining, disease outbreaks, climate change, rodenticide poisoning [110], culling of barred owls [111], shifts of wildfire regimes from climate change, and many other factors that may affect population performance. Our generalized approach is flexible enough to accommodate relatively simple scenarios like we used or far more nuanced and sophisticated forecasting such as predicting climate change impacts to habitat, prey, and competitors in a spatially explicit manner. We would also recommend that other researchers evaluate many different candidate networks (size and distribution) as well as scenarios (assumptions about habitat, prey, competitors, etc.). Doing so provides decision makers with more “option space”, given insights into how simulated populations respond to variation in network size and distribution, and assumptions about important ecological conditions.

Different portions of the landscape were prioritized when using resource selection function (RSF) maps versus when HexSim was used in combination with RSF maps to model biologically-informed population outcomes for Greater Sage-Grouse (*Centrocercus urophasianus*) [112]. HexSim was also used to evaluate potential effects of renewable energy development on Golden Eagles (*Aquila chrysaetos*) [113]. In those studies and ours, insights that were not possible or obvious without the modeling tools helped to identify conservation actions more likely to result in positive population outcomes. Nonetheless, it is important to understand and identify key model assumptions and how influential they are on model outcomes. For example, our sensitivity analyses of the HexSim spotted owl model showed that population outcomes were highly sensitive to variation in adult survival. For the spotted owl, it has long been known that populations are most sensitive to adult survival [103]. Furthermore, empirical estimates of adult survival exist for many study areas throughout the owl’s range [38, 39], so we were confident with the values we used in the model.

We could have used our RHS maps to quantify NSO population dynamics through the construction of patch dynamics models (e.g., metapopulation or patch occupancy models). While effective pedagogic tools, patch dynamics models have limited practical utility compared to the HexSim simulator. Metapopulation and patch occupancy models, for example, extrapolate measures of population size and stability from estimates of patch-specific colonization and extinction rates, but do not account for within-patch dynamics, nor can they accommodate movement behaviors or impacts of disturbance exhibited by some individuals but not others. And, unlike the parameters informing our mechanistic IBM (e.g., biological upper-limits on vital rates), extinction and colonization rates cannot be assumed immutable. Hence, patch dynamic models are more constrained regarding population forecasting. The rationale for developing patch dynamics models is that they can account for spatial pattern while having minimal data requirements, and this may be a good reason for their use in some circumstances. But the colonization and extinction rates they require are typically not well characterized, nor is it clear that distinct resource patches can always be identified within our RHS map. In contrast, our parsimonious but mechanistic IBM did not necessitate tessellating the RHS map, and it was based on life history information that has been recorded consistently for decades over much of the NSO's range. Patch occupancy and metapopulation models set the stage generally for the development of spatial IBMs, but the latter now constitute the best available science, and as such are sought out by regulatory agencies. Plus our spatial IBM can be made more biologically nuanced as additional demographic information becomes available, as illustrated by previously cited efforts to extend its utility through the addition of a full barred owl population sub-model.

### Model estimates and reality

As with several previous efforts at devising conservation plans for the spotted owl, we attempted to use and synthesize the extensive empirical data available. Conservation planners recognize that the models they use are imperfect, and often rely on proxies for environmental features that have no valid estimates, or when those estimates don’t exist in a spatially explicit way. The ultimate measure of a conservation plan is its effectiveness [15]–a measure that is only available over some (generally long) time period. Nonetheless, and notwithstanding the uncertainties inherent in these processes, confidence in model outputs can and should be tempered relative to their concordance with contemporary empirically-derived estimates.

Our models were largely informed by empirical research on spotted owls. Our RHS models were based primarily on published studies, but some variables included were informed by expert opinion. Nonetheless, the resulting RHS predictions showed strong fidelity to empirical studies of spotted owl habitat selection. That is, high RHS values within each modeling region corresponded to areas with more basal area of large-diameter trees, high percentage canopy cover, etc. (B. Woodbridge and J. Dunk, unpublished data). From RHS estimates, we estimated “resource value” for each territorial owl, an over-arching feature that represented the sum total of resources that we did not have spatially explicit estimates of. Because of the well-established variation in spotted owl home range size, we varied resource targets among modeling regions by a factor of 5 (Table E in S1 Appendix). Age specific survival estimates from the meta-analysis that occurred nearly coincident with our analyses [38] were also used. We set three classes of age-specific survival as a function of the percentage of the target resource value owls acquired in their home ranges. Empirical studies informed, but did not directly guide, our decision making process. For example, survival (and fitness) were related to spotted owl habitat characteristics [7]. Our division of age-specific survival into three resource classes was logical, but one of many possible choices that could have been made. Ultimately, the question is whether the myriad decisions made in developing the model(s) resulted in realistic population-level patterns of distribution, movement, and abundance. Our model estimates of owl density were close to empirical estimates for six of eight demographic study areas we found data for (S1 Fig). For the remaining two, one model estimate was too high and the other too low. Similarly, estimates of dispersal distances between modeled and real owls showed very similar distributions (S2 Fig).

In the meta-analysis of the finite rate of population change [38], the top-ranked *a priori* model included ecoregion and proportion of spotted owl territories with barred owl detections. In our modeling, we used estimates of barred owl encounter rates from DSAs [38], and applied them to our modeling regions. In an effort to further evaluate the proxies we chose in our modeling, we overlaid modeling region boundaries with DSA boundaries and estimated mean RHS of each DSA by modeling region subdivision (n = 18). We then created a generalized additive regression model using mean RHS and barred owl encounter rates to predict finite rate of population change from [38]. For the DSAs that overlapped more than one modeling region, the same estimated lambda value from [38] was used in both regions. We then evaluated the relationship between our estimated lambda, based on RHS and barred owl encounter rate, and the DSA lambda values from [38]. The coefficient of determination between the two was 0.81 (of the 18 differences, the *largest* absolute value of difference between the two estimates was 0.0197). The value of this exercise was to evaluate whether the time-consuming data gathered by many scientists over the course of, sometimes, multiple decades and analyzed using sophisticated mark-recapture techniques could be closely predicted using our proxies. Importantly, our estimates would have no known mooring to reality without these empirically-derived estimates. This specific evaluation may also provide insights into what, among the ecoregions, within the top-ranking model of [38] was driving the relationship; perhaps variation in RHS. Although approached differently, the findings of [38, 40, 50, 51], and ours all suggest that habitat and barred owls are important drivers of spotted owl distributions, abundances, and population dynamics. In the revised recovery plan for the spotted owl [45], it was noted that critical habitat was necessary, but not sufficient alone, to recover the owl. A project related to ours [102] reached the same conclusion. Our analyses corroborate this contention. We suggest that the close correspondence between our models’ estimates and empirical data meets the “realism” standard for informing conservation planning.

The most conservative interpretation of model outputs is to treat them as purely relative to other scenarios, such that some scenarios have better or worse population outcomes than others. However, we strove to develop a model that was also accurate. Due to the closeness of model estimates and empirical data, our model estimates can also be used to make more nuanced statements about the degree of difference among various scenarios. That is, rather than “better or worse”, our modeled population performance metrics can also be interpreted to represent how much better or worse owls would be expected to perform under various scenarios.

Given the purpose for which our modeling framework was developed, the application of the models for other purposes has some limitations. For example, the models were developed for use and tested at relatively large spatial scales. We do not have a specific area threshold below which it would be inappropriate to use the model but we recommend that it be used at relatively large scales. For example, a national forest could use our HexSim model to evaluate the estimated effects of potential future management actions or other changes to their lands on spotted owls. However, we would also recommend that the models should be run on much larger modeling region or range-wide landscapes than the national forest is embedded within. For these exercises, we recommend either holding other landscape areas constant or making assumptions about what may happen to spotted owl habitat on other ownerships over time. HexSim allows reports for any particular geographic area to be generated, so evaluating what happens to simulated owls on relatively small areas among various scenarios can occur when applying the model to a large area. We strongly caution against the application of our HexSim model to small landscapes that are treated in an insular way (i.e., treating a study area or ownership as if it exists in a vacuum).

### Conservation and management implications

Although we conducted a time- and labor-intensive process to develop and evaluate candidate networks, one of which (Composite 11) was chosen and designated as critical habitat [105], we do not believe this was the most important part of the “critical habitat” process. The most important part of the process is what comes next; in deciding what happens where and when within critical habitat and monitoring the effects of those decisions. The Revised Recovery Plan for the Northern Spotted Owl [45] and the final rule designating critical habitat [105] recommended maintaining and restoring high quality spotted owl habitat and active management within critical habitat. Hence, the intentions of the USFWS were to maintain the most valuable areas for the owls but allow management to restore, improve, or protect other areas.

Our findings could be used to identify areas to provide strong protections against habitat loss (e.g., ≥50 RHS) as well as identifying lower suitability areas (e.g., <30 RHS) where various management treatments, including ecological forestry approaches [114] might be warranted. Such management approaches could have the goal of protecting high suitability areas for spotted owls and other species with overlapping habitat associations and restoring or improving habitat conditions, reducing fire risk, or generating revenue in low suitability areas. For areas of intermediate suitability (RHS 30–49.99), a more case-by-case approach to the management is warranted. In general, active management within critical habitat should consider the larger landscape including the amounts and spatial patterns of habitats of all suitability levels. In some portions of the owl’s range, intermediate RHS areas may represent the best conditions for owls. Such circumstances may warrant greater protection. In other portions of the range, intermediate suitability areas may coincide with high wildfire risk and hazard, and be reasonably targeted for thinning, especially when thinning those areas provides a protective benefit to nearby high-suitability areas.

Barred owl populations within the range of the spotted owl are apparently not currently at equilibrium. There is evidence of increases in barred owl populations [39], since the work of [38] that we used to parameterize our models. Hence, it is likely that barred owl encounter rates, and thus impacts, in many portions of the spotted owl’s range will increase if no countervailing management actions are taken. Our models that assumed a barred owl encounter probability of 0.5 throughout the range resulted in estimated spotted owl population declines by about 90%. Declines of that magnitude would almost certainly lead to the disappearance of the spotted owl from large portions of its currently-occupied range, if not functional extinction. Using the spotted owl HexSim model presented herein, barred owl management scenarios (encounter probability decreased to 0.15) within accessible (by road) high suitability conservation lands in Washington state were evaluated and spotted owl population responses compared to scenarios with no barred owls and with no barred owl control (0.5 encounter probability state-wide) [115]. Under the no barred owl control scenario state-wide owl populations declined by 95%, but under the barred owl control scenario populations declined by about 50%. Other, more nuanced, and spatially explicit scenarios can be evaluated, perhaps with implementation costs (e.g., see [116]) or other socio-political constraints included. In the redwood region, on-the-ground barred owl removal resulted in large differences in spotted owl population trends (2.9% increase) in areas where barred owls were removed compared to areas where they were not removed (13% decrease) [117]. Improvements in our modeling process, such as moving from forecasting scenarios to making predictions about future conditions could be accomplished with more realistic and spatially-explicit estimates of forest growth, wildfire, and harvest throughout the spotted owl’s range. Similarly, a two-species HexSim model that included both spotted owls and barred owls, and that modeled interspecific competition, would also likely add realism to such predictions.

Our process of synthesizing the vast literature on spotted owls and incorporating it into modern conservation planning approaches (species distribution modeling with MaxEnt, reserve location modeling with Zonation, and population modeling with HexSim) provides a repeatable and scientifically defensible approach to evaluating many candidate networks and alternative scenarios. Doing so allows decision makers a better understanding of risk and the potential for successful conservation. Additionally, our approach is modular, such that more information can be incorporated into the modeling approaches we developed. For example, more nuanced habitat change information or a two-species population model can be incorporated into the models we’ve developed. The majority of species of conservation concern do not have the wealth of information that we had for the spotted owl. Nonetheless, our generalized modeling approaches can be utilized for many species, and new information can be incorporated as it becomes available. Thus, the modeling and decision making framework can and should be adaptable to new information.

## Supporting information

S1 Appendix Details of modeling methods and results. (DOCX)

S1 Fig Differences between empirical estimates of northern spotted owl densities and HexSim-derived estimates (mean and 95% CI of 5 replicates) for eight demographic study areas. (TIF)

S2 Fig Comparison of distribution of northern spotted owl dispersal distances derived empirically from banded owls (Forsman et al. 2002; n = 328) and from HexSim simulations (n = 850,000). (TIF)
