# Supplementary material for: Conservation planning for species recovery under the Endangered Species Act: A case study with the Northern Spotted Owl
Source: PLoS One. 2019 Jan 14;14(1):e0210643. doi: 10.1371/journal.pone.0210643 (PMC6331132; doi:10.1371/journal.pone.0210643)
Supplement: S1 Appendix — (DOCX) [file pone.0210643.s001.docx]

**Methods**

**Spotted owl site center locations**

We compiled a large sample of site centers broadly representative of the spotted owl’s geographic range. However, the distribution of site centers in our original sample was spatially biased. DSAs provide the highest-quality data sets, but they comprise only about 9% of the spotted owl’s geographic range [41]. Because the DSAs have been surveyed intensively over time, data from these areas are generally spatially aggregated. Outside of DSAs, survey effort was not consistent and the quantity and density of spotted owl location data varied widely and included areas with few site centers. Areas with few site centers could occur due to the absence of spotted owls, or from few surveys being conducted, or data being unavailable to us. Spatially biased survey data present major challenges to distributional modeling by over-weighting areas where intensive sampling has occurred [62]. Therefore, within each modeling region we “thinned” the spotted owl locations from all data sources such that the minimum distance between them would be 3 km. Using a 3 km thinning distance retained approximately 75% of the original locations (Table A). We reasoned that this distance represented a balance between spatial survey bias and real on-the-ground differences in densities of site centers.

**Table A. Sample size of spotted owl site center locations (1993-1999) by modeling region and the impact of various thinning distances (minimum allowable distance between site centers) on sample size.**

|  | | **Thinning Distance** | | | | | |
| --- | --- | --- | --- | --- | --- | --- | --- |
| **Modeling**  **Region** | **Total Sites** | **1 km** | **1.5 km** | **2 km** | **2.5 km** | **3 km** | **4 km** |
| **NCO** | 241 | 236 | 229 | 221 | 209 | 196 | 162 |
| **OCR** | 454 | 430 | 414 | 371 | 325 | 281 | 202 |
| **RDC** | 724 | 716 | 670 | 547 | 461 | 392 | 284 |
| **WCN** | 80 | 80 | 79 | 78 | 77 | 77 | 74 |
| **WCC** | 214 | 211 | 205 | 195 | 182 | 173 | 144 |
| **WCS** | 489 | 489 | 487 | 482 | 477 | 470 | 342 |
| **ECN** | 216 | 215 | 209 | 203 | 195 | 184 | 155 |
| **ECS** | 123 | 122 | 119 | 112 | 104 | 93 | 67 |
| **KLW** | 462 | 460 | 454 | 440 | 414 | 358 | 275 |
| **KLE** | 472 | 468 | 463 | 455 | 434 | 381 | 285 |
| **ICC** | 308 | 308 | 307 | 300 | 286 | 253 | 199 |
| **Total** | **3783** | **3735** | **3636** | **3404** | **3164** | **2858** | **2189** |
| **Percentage of total** | **100** | **98.7** | **96.1** | **90.0** | **83.6** | **75.5** | **57.9** |

**Generalized Nearest Neighbor (GNN) data**

Forest attributes from inventory plots (e.g., Forest Inventory and Analysis, Current Vegetation Surveys) were imputed to map pixels based on modeled relationships between plots and predictor variables from Landsat thematic mapper imagery, climatic variables, topographic variables, and soil parent materials. For the NWFP Effectiveness Monitoring program, GNN maps were created for the two “bookend” time periods (1996 and 2006 in Oregon and Washington, 1994 and 2007 in California). The GNN maps come with a large suite of diagnostics detailing map quality and accuracy; these are contained in region-specific accuracy assessment reports available at <http://www.fsl.orst.edu/lemma/>.

For developing *a priori* models of spotted owl habitat, we generally selected GNN structural variables with plot correlation (between ground-measured and GNN-predicted) coefficients >0.5 for each modeling region (42% were >0.7; see 48, Table C-1). For species composition variables, we generally used only variables with Kappas >0.3 (see [48], Table C-2). Cohen’s Kappa measured the accuracy of field versus GNN-predicted composition variables relative to agreement expected by chance. The GNN vegetation database was specifically developed for mid- to large-scale spatial analysis [60], suggesting that accuracies at the 30-m pixel scale may be less influential to results obtained at larger scales [65]. Because we were interested in the utility of GNN at our analysis scale (200-ha, we compared the distribution of GNN variable values at a large sample of actual locations (known spotted owl nest and roost sites and foraging sites) to published estimates of those variables at the same scale. In addition, we compared GNN maps to a number of local plot-based vegetation maps prepared by various field personnel.

**MaxEnt modeling of telative habitat suitability**

The MaxEnt modeling process was as follows:

1. To determine each modeling region’s best NR habitat definition we compared all within-region NR definitions for that region to each other.
2. We combined the best NR habitat definition(s) with each modeling region’s foraging (F) habitat definition to evaluate whether the addition of foraging habitat improved model performance. Models were considered to have been improved if the addition of F habitat increased the ranking of the model. If the addition of F habitat improved the model’s performance, we used the NR + F habitat model for step 3 (below). If not, we used the best NR model(s) for step 3.
3. Using the best model from step 2, we evaluated model improvement by considering core, edge, and core + edge covariates. The best model was carried forward.
4. Dominant tree species composition variables were then evaluated based on the best model from step 3. Each compositional variable was entered sequentially such that more than one could end up in the model that was carried forward.
5. The best model from step 4 was evaluated by considering the inclusion of abiotic covariates such as landscape curvature, elevation, and relative position index.
6. The best model from step 5 was evaluated by adding climate covariates.

**Interpreting MaxEnt models**

AUC values describe the proportion of times one could expect a random selection of a spotted owl nest site location to have a larger RHS value than a random selection from available locations. It is a threshold-independent measure of model discriminatory ability. Because our evaluation does not contrast use versus non-use, AUC values have an upper limit less than 1.0 (because some of the available locations are actually used by spotted owls). Even for good/excellent (well-discriminating) models, AUC values should be lower in areas where the landscape contains larger amounts of suitable habitat. Although AUC is a measure of discrimination, a use-versus-availability model’s ability to discriminate is a function of both the strength of habitat selection and the abundance of suitable habitat. In order to evaluate the degree to which AUC values were a function of the amount of suitable habitat in modeling regions, we evaluated the correlation between AUC values and the percentage of each modeling region with RHS scores above several thresholds.

**RHS changes for optimistic and pessimistic scenarios**

For the optimistic RHS change scenario, we applied the “observed” gains and losses in RHS as follows: *within networks*, future gains and losses in RHS occurred as estimated from 1996-2006, whereas *outside of network* gains were reduced by 50% and losses occurred as observed. In most circumstances the outside of network RHS changes resulted in a small net decrease of RHS. We believed this was a reasonable assumption about future forest management on non-network lands given the forest management practices that have been applied on these lands in recent decades [66]. For the six percentage change classes, we projected the midpoint of each of the first five classes, which were ranges, but for the >5% class we estimated the mean amount of change that was estimated between 1996 and 2006 for each RHS category for each modeling region, and projected that value. The optimistic scenario was implemented as two 20-year change increments (RHS changes inserted at time steps 70 and 90), compounded (i.e., two steps at 10% each, not one step at 20%). Hexagons to change were randomly selected, with replacement.

**Zonation**

Zonation essentially works by iteratively removing each cell from the landscape, using minimization of marginal loss as the criterion to decide which cell is removed, until no cells remain [98]. Minimization of marginal loss is the iterated removal of cells, such that each removal results in the smallest decrease in the conservation value of the remaining network [98]. Zonation records the order of cell removal, allowing the user to then select the most valuable fraction of the modeling area for retention (e.g., top 30%, 40%, or 50%; [98]).

Zonation has several species-specific settings that can influence its output. Zonation requires the identification of species-specific factors such as dispersal capabilities and response to habitat fragmentation to rank each map pixel’s value and can include constraints to possible solutions (networks) based on patterns of land ownership. Variation in spatial reserve design produced by Zonation reflect user-defined scenarios that we evaluated and compared. The specific settings we used in Zonation were tuned to be relevant to the northern spotted owl.

Zonation allows for the choice of efficient conservation solutions. Efficient means the smallest area that meets the conservation objectives, so as to maximize the habitat value per unit area within a network (see Fig 7 in Appendix C of [48]). For example, in one of the Zonation scenarios we evaluated, 70% of the habitat value existed on approximately 40% of the landscape.

**The HexSim model**

Our HexSim spotted owl scenario was a females-only model because female owls are the most influential sex in terms of population dynamics [103]. The simulated acquisition of resources by individual spotted owls was spatially stratified by modeling region. The scenario depended on two static spatial data layers (maps); one representing the range extent and RHS, and an additional layer identifying the boundaries of the 11 modeling regions (the modeling regions were both a model input – affecting home range size and resource availability, and were used to organize model output). MaxEnt RHS model results were at 30-m resolution, but each 30-m cell’s value was a function of the conditions within 200-ha of it. The 30-m resolution MaxEnt RHS data were converted to 86.6-ha hexagon grids in HexSim representing mean RHS scores of all 30-m pixels within each hexagon. To the extent possible, simulation parameter values were estimated based on published empirical data. More details of the HexSim spotted owl model can be found in [45, 102, 104].

Five traits were created as part of the spotted owl population definition:

1. Stage class. Simulated spotted owls began life at age zero, and stage class zero. Each year they survived they transitioned into the next stage class. At age 3 they reached stage class three, which was the terminal stage class.
2. Resource class. Each owl always belonged to one of three resource classes, depending on the amount of resources they were able to acquire from their home range. Spotted owls that acquired 2/3 or more of their resource target (see below) were placed in the high resource class, and those that attained less than 1/3 of their resource target were placed into the low resource class, while all other spotted owls were placed into the medium resource class. Resource targets varied by modeling region (see below).
3. Territory status. The territory status trait was used to record whether individual spotted owls owned a territory or were non-breeding floaters.
4. Barred owl exposure class. The barred owl presence trait categorized individual spotted owls as being exposed, or unexposed, to a barred owl.
5. Location (modeling region). Modeling region traits were used to track individual spotted owl locations. The 11 modeling regions were space-filling and non-overlapping. If a spotted owl territory spanned multiple modeling regions, it was assigned to the region in which the majority of its territory hexagons fell.

We initiated HexSim simulations with 10,000 spotted owls being placed into the virtual RHS landscape, constituting a "free response" model condition of initially overpopulating the region and then letting the dynamics of individual movements and settlements subside into equilibrium after the initial start-up bias period [102]. We preferentially placed owls into areas of high mean RHS. The initial population's ages were randomly distributed. Simulated owls then attempted to construct a territory and subsequently acquire resources within their home ranges. We used the RHS map as a proxy for resources available to spotted owls within each hexagon. Because actual breeding spotted owls showed relatively strong selection for some RHS categories and against others, we reasoned that this selection/avoidance was based on a combination of factors that influence spotted owl fitness. We assumed that spotted owls select some areas and avoid other areas in order to maximize their survival and reproductive success, within the constraints of what is available to them and the occupancy and use of areas by other spotted owls. Spatially-explicit data on prey and predators were unavailable, and thus we did not incorporate these factors into our model beyond the RHS values that implicitly include them as a component of habitat suitability.

In the HexSim spotted owl scenario, a primary influence of RHS on simulated spotted owl populations occurs in territory acquisition. To the extent that some areas are not selected by spotted owls (or disproportionately selected against), RHS acts to limit survival and reproduction. That is, spotted owls don’t survive or reproduce in areas that they do not occupy. Subsequent to territory establishment, resource acquisition within home ranges determined the resource class a spotted owl was placed in, which influenced survival rates. Reproduction was not influenced by resource acquisition, and thus was not influenced by RHS, other than through dictating whether an area was at least minimally suitable for occupancy. Individual studies [7] and meta-analyses have reported influences of habitat on survival and in some cases fecundity [38].

Although we did not define or attempt to model dispersal habitat explicitly, we made assumptions about the influence of RHS on successful dispersal. Success (survival) of spotted owls dispersing through variable landscapes may be influenced by factors similar to those affecting territorial spotted owls (e.g., availability of prey, cover from predation, thermal stress), albeit at different scales. In our HexSim model, spotted owls possessed moderate forward momentum and were allowed to disperse through the full range of RHS values, though they tended to avoid low RHS areas. Specifically, simulated dispersers were assigned an autocorrelation coefficient with values of roughly 0.02, 0.08, 0.45, and 0.45 to hexagons that fell directly behind, behind to the left or right, ahead to the left or right, and directly ahead of their present location, respectively. Dispersers also assigned neighboring sites an avoidance coefficient that ramped linearly from 0.1 in hexagons with an RHS of 0, to 1.0 in hexagons with an RHS of 30 or more. A rescaled product of the forward movement and avoidance coefficients set the likelihood that each hexagon surrounding a disperser would be entered during its next movement step.

The simulated spotted owls had territory sizes of no more than three 86.6-ha hexagons. This territory size represents a reasonable approximation of a spotted owl core area (200-ha). Individual hexagons had to have at least a mean RHS score of 35 (out of 90 possible) to be usable in forming a territory. We chose a minimum score of 35 after evaluating the scores of hexagons overlaid on 3,790 known spotted owl site centers. We evaluated the score for the focal hexagon (the one in which the site center resided), the second, and third closest hexagons, as well as the mean scores of the first, second, and third hexagons. More than 75% of the site centers were in hexagons with scores ≥35. Similarly, 73% of the spotted owl sites had a mean score >35 for the focal, second, and third closest hexagons. Although other scores might be valid, we reasoned that increasing the score would inhibit settlement on empirically suitable areas, whereas decreasing the score would likely result in unrealistic densities in areas with relatively low RHS. Territory size had little significance for the simulated population dynamics, as the spotted owls derive resources from their home ranges. The territories essentially served as a core area around which home ranges were constructed.

After colonizing a territory, each simulated spotted owl established a home range. The simulated spotted owls had small defended territories (or core areas; [7-9, 41, 79-81]), but large overlapping home ranges. Home range size varied with modeling region (Table B) and was set to the mean of the available region-specific estimates (see summary in [105]). Spotted owls “acquired” resources from their home ranges, and conspecific competition was simulated by evenly dividing the resource value of a hexagon among the number of spotted owls whose home ranges it was included in. For each owl, resource acquisition was represented as the total value of hexagons within its home range.

**Table B.** **Resource targets (summation of relative habitat suitability values) used in HexSim, based on relative habitat suitability values at 3,790 northern spotted owl locations.**

| **Modeling Region** | **Home Range Size**  **ha (# hexagons)** | **Resource**  **Target** |
| --- | --- | --- |
| North Coast Olympics | 11,052 (128) | 1250 |
| East Cascades North | 7,258 (84) | 1000 |
| West Cascades North | 7,258 (84) | 1250 |
| West Cascades Central | 7,258 (84) | 1250 |
| Oregon Coast | 4,123 (48) | 375 |
| West Cascades South | 3,949 (46) | 375 |
| Inner CA Coast Range | 3,165 (37) | 375 |
| East Cascades South | 3,033 (35) | 750 |
| Klamath East | 3,033 (35) | 375 |
| Klamath West | 3,033 (35) | 375 |
| Redwood Coast | 1,173 (14) | 250 |

After simulated spotted owls finished prospecting for territories, the modeling region they were in was recorded. The determination of whether each territorial spotted owl was in the presence of a barred owl was then made probabilistically, depending on the modeling region (Table C). The region-specific probabilities for spotted owls encountering a barred owl were based on the proportion of spotted owl territories where barred owls were detected each year on the 11 DSAs (see Appendix B in [38]). More directly accounting for barred owl impacts on spotted owl habitat selection or site fidelity would require that barred owls be located in a spatially explicit manner on the simulated landscape, and possibly even fully simulated within HexSim. At the time we developed the HexSim spotted owl model, insufficient data existed range-wide to permit either option to be incorporated into the simulations. The finest level of resolution we felt was reasonable was to include region-specific barred owl encounter probabilities for territorial spotted owls. Whether or not a barred owl was present was only decided once per “bird-territory” (i.e., once the decision was made for an individual spotted owl at a territory, the barred owl presence/absence was fixed for that territory until another spotted owl took over the territory).

**Table C. Probability of a territorial northern spotted owl encountering a barred owl in its territory among modeling regions; estimated from Forsman *et al.* (2011).**

| **Modeling Region** | **Encounter**  **Probability** |
| --- | --- |
| North Coast Olympics | 0.505 |
| East Cascades North | 0.296 |
| West Cascades North | 0.320 |
| West Cascades Central | 0.320 |
| Oregon Coast | 0.710 |
| West Cascades South | 0.364 |
| Inner CA Coast Range | 0.213 |
| East Cascades South | 0.180 |
| Klamath East | 0.245 |
| Klamath West | 0.315 |
| Redwood Coast | 0.205 |

Spotted owls were given one of 24 annual survival rates depending on stage class, resource acquisition class, and exposure to barred owls (Table D). To begin with, survival rates (estimated apparent survival) for stage class 1-3 spotted owls were derived from Table 12 in [38]. Because true survival is unknown, we made the assumption that apparent survival is equal to, or a reliable surrogate for, true survival. Stage class-specific survival estimates were provided for each of 11 DSAs [38]. For each study area and stage class, mean apparent survival values for males and females were provided. We computed the mean of each pair and identified the smallest and largest of these mean values. Stage class 0 survival estimates were taken from [36]. We used the mean, minimum, and maximum stage class 0 survival rates. The mean value for stage class 0 was set to the midpoint between the minimum and maximum value. For any given stage class, the smallest mean value was assigned to individuals in the low resource class. Likewise, the largest stage-specific mean value was assigned to individuals in the high resource class. The stage-specific survival rates for individuals in the medium resource class were set equal to the mean taken over all of the survival estimates present in Table 12 of [38], or [36], for that stage class. Through this process survival rates were obtained for stage 0-3 spotted owls in all three resource classes. We adjusted survival rates for the impact of barred owls across all study areas, as calculated from the survival meta-analysis model containing an additive barred owl effect [38]. Each spotted owl was either impacted by a barred owl, or not impacted. Hence, the NSO HexSim model contained 24 survival rates (4 stage classes x 3 resource classes x 2 barred owl impact categories; Table D). Barred owls only affected territorial spotted owls in our simulations, so all floaters were necessarily free from such impacts. At the time the survival decision was made, juvenile spotted owls had dispersed, but not yet had an opportunity to prospect for a territory. Thus, our simulated juvenile spotted owls were never exposed to barred owl impacts, but all of the older age classes could have been subjected to this stress.

**Table D. Survival rates of northern spotted owl based on stage class, resource class, and barred owl effect used to parameterize HexSim.**

| **Without Barred Owls** | | |  | **With Barred Owls** | | |
| --- | --- | --- | --- | --- | --- | --- |
| **Stage**  **Class** | **Resource**  **Class** | **Survival**  **Rate** |  | **Stage**  **Class** | **Resource**  **Class** | **Survival**  **Rate** |
| Stage 0 | Low | 0.366 |  | Stage 0 | Low | 0.28 |
|  | Medium | 0.499 |  |  | Medium | 0.413 |
|  | High | 0.632 |  |  | High | 0.546 |
| Stage 1 | Low | 0.544 |  | Stage 1 | Low | 0.458 |
|  | Medium | 0.718 |  |  | Medium | 0.632 |
|  | High | 0.795 |  |  | High | 0.709 |
| Stage 2 | Low | 0.676 |  | Stage 2 | Low | 0.590 |
|  | Medium | 0.811 |  |  | Medium | 0.725 |
|  | High | 0.866 |  |  | High | 0.780 |
| Stage 3 | Low | 0.819 |  | Stage 3 | Low | 0.733 |
|  | Medium | 0.849 |  |  | Medium | 0.763 |
|  | High | 0.865 |  |  | High | 0.779 |

Spotted owl fecundity (females produced per female) rates varied by stage class (Table E), and were based on data in Table 3 of [38]. Barred owls now occupy nearly all of the spotted owl’s range [39] but their numbers (represented as encounter probabilities herein) likely vary geographically. Thus, while we did not explicitly model barred owl effects on spotted owl reproduction, those effects were implicitly incorporated into our modeling through our use of published fecundity rates from populations impacted by barred owls.

**Table E**. **Northern spotted owl fecundity (females produced per female) by stage class used in HexSim model (taken from Forsman et al. 2011).**

|  |  | | | |
| --- | --- | --- | --- | --- |
|  | **Fecundity** | | | |
| **Stage Class** | **0** | **1** | **2** | **Mean for Stage Class** |
| **0** | 1 | 0 | 0 | 0 |
| **1** | 0.953 | 0.023 | 0.023 | 0.070 |
| **2** | 0.865 | 0.067 | 0.067 | 0.202 |
| **3** | 0.780 | 0.110 | 0.110 | 0.330 |

The baseline HexSim spotted owl scenario used three different map files: 1) RHS, 2) modeling region boundaries, and 3) demographic study area boundaries. The demographic study area map was only used for reporting purposes, but the modeling region map was also used to adjust resource acquisition and determine home range size. All three maps were static (their boundaries did not change with time), and each was comprised of 543,400 hexagons arranged in 1,430 rows and 380 columns. Individual hexagons were 1,000 m in diameter, and had an area of 86.6-ha. In HexSim, each RHS pixel was assigned a weight equal to its RHS score. Pixel scores ranged between zero and 97. Thus, when the HexSim RHS map was constructed from the 30-m resolution MaxEnt map, the largest possible hexagon score was 97.00; this upper limit was never realized because each hexagon’s value represented an average of the pixels within it. The RHS scores of hexagons in the HexSim RHS map ranged from 0.00 to 90.37.

From ([38], Table 12) we calculated the mean variation as the ratio of 2·SE of adult survival divided by the mean adult survival. For each of the DSAs for which data were presented, mean variation was 2.59% (range = 1.64–4.52%). We used 2.5% variation in survival rates for each age class, even though separate calculations for younger age classes would have been much greater [41]. The temporal process variation for adult owls, however, would likely have been lower than 2.5%. Nonetheless, 2.5% variation was a reasonable compromise to apply to all age classes and it was applied across all networks and scenarios, so the impact of an over or underestimate of actual rates still allows for meaningful comparisons of spotted owl population responses among various networks and scenarios. The 50% variation in fecundity was an attempt to allow for the wide variation in annual reproduction that has been observed in many spotted owl population studies [38, 39].

When the stochastic simulations were run, one member from the following set was selected at random each year to determine survival for that year. If the collection of survival rates shown in Table D are placed into a vector S, then the family of survival rates used in the stochastic simulations could be represented as the set {0.975 · S, 0.980 · S, 0.985 · S, 0.990 · S, 0.995 · S, S, 1.005 · S, 1.010 · S, 1.015 · S, 1.020 · S, 1.025 · S}. This set has 11 members, five of which represent survival rates lower than those in S, and five that represent survival rates higher than those in S.

When the stochastic simulations were run, one member from the following set of 3 elements was selected at random each year to determine fecundity by age class for that year. If the collection of fecundities without environmental stochasticity (Table E) are placed into a vector F, then the family of reproductive rates used in the stochastic simulations could be represented as the set {0.5 · F, F, 1.5 · F}.

**HexSim sensitivity analyses**

Our sensitivity analysis included 21 additional HexSim simulations, each consisting of 100 replicates (Table F). The nine parameters we modified were: 1) the minimum value for a hexagon to be included in a spotted owl’s territory; 2) the minimum resource necessary to form a territory; 3) the modeling region-specific resource targets; 4) the threshold values defining the low, moderate, and high resource acquisition categories; 5) the maximum explored area; 6) survival rates; 7) reproductive rates; 8) the resource quality goal used within dispersal; and 9) the extent to which poor quality hexagons are avoided by dispersing owls. Based on demographic analyses of spotted owls [38-40], we expected modifications of survival rates to have disproportionately large effects on population sizes, and variation in reproduction to have relatively less impact. Furthermore, we expected the threshold RHS value of a hexagon that was deemed suitable for territory establishment (35 in our base-model setting) to also have a relatively large impact on population performance. We used a constant RHS environment and had no environmental stochasticity for the sensitivity analyses. For each modeling region and range-wide, we calculated the ratios of mean population size at time step 350 from 100 replicates of models with each perturbed parameter to the base model’s mean population size at time step 350. Ratios close to 1.0 suggest little effect of that parameter change on the model results, whereas values appreciably greater or less than 1.0 suggest stronger effects (larger or smaller populations) of that parameter.

**Table F. Parameters modified for sensitivity analysis of northern spotted owl HexSim Model. The first column is a parameter identification value, and the last four columns (A-D) represent the specific values assigned to a parameter. Each parameter-value pair corresponds to a specific 100-replicate simulation. For example, scenario 6A was used to quantify the impact on population size resulting from a 2.5% reduction in survival rates (across all stage class x barred owl categories).**

|  |  |  |  | A | B | C | D |
| --- | --- | --- | --- | --- | --- | --- | --- |
|  | **HexSim Modeling Section** | **Parameter** | **Value used in base NSO HexSim Model** | **low1** | **low2** | **high1** | **high2** |
| 1 | Population > Range Data | Hexagons Range-Eligible if Value at Least | 35 | 30 |  | 40 |  |
| 2 | Population > Range Data | Minimum Range Resource | 105 | 95 |  | 115 |  |
| 3 | Population > Range Data | Resource Targets within modeling regions |  | -10% | -25% | 10% | 25% |
| 4 | Population > Traits > Resource Class | Resource trait threshold |  | 25, 50 |  | 25, 75 | 50, 75 |
| 5 | Event Sequence > Movement > Floater Prospecting > Exploration | Maximum number of hexagons explored | 500 | 400 |  | 600 |  |
| 6 | Event Sequence > Survival > Stage Class | Rates: specified by stage class under normal nesting | varied by stage class, resource acquisition class, and barred owl presence | -2.50% |  | 2.50% |  |
| 7 | Event Sequence > Reproduction > Stage Class | Rates: specified by stage class under normal nesting | varied by stage class | -10.00% |  | 10.00% |  |
| 8 | Event Sequence > Movement > Stage 0 Dispersal > Dispersal | Mean Resource Quality | 35 | 30 |  | 40 |  |
| 9 | Event Sequence > Movement > Stage 0 Dispersal > Dispersal | Repulsion maximum | -3.3 | -1.5 |  | -5 |  |

**Evaluating RHS models with independent site center locations**

We calculated the change in mean RHS at the hexagon scale in each modeling region for five categories of RHS, and the direction of change. The five RHS categories for which we estimated RHS change were: 0-20, 20-40, 40-60, 60-80, and >80. We also created six categories reflecting actual RHS percentage change between 1996 and 2006: <1%, 1-2%, 2-3%, 3-4%, 4-5%, and >5%. We estimated the percentage of each modeling region within each RHS category that increased in RHS and that decreased in RHS, by each RHS percentage change class.

**Results**

Table G. Percent contribution of covariates in each modeling region’s MaxEnt relative habitat suitability model.

| **North Coast / Olympics** | | **Oregon Coast Ranges** | | **Redwood Coast** | |
| --- | --- | --- | --- | --- | --- |
| **Full Model** | **%** | **Full Model** | **%** | **Full Model** | **%** |
| NR 06 | 42.4 | NR 08 | 29.4 | Slope Position | 48.2 |
| NR06Edge | 21.5 | NR08 Edge | 24.2 | Curvature | 11.2 |
| NR06+F04 | 20.1 | Slope position | 11.9 | NR03 Edge | 10.3 |
| Slope position | 6.0 | July Max Temp | 10.1 | NR03 + F05 | 6.1 |
| Elevation | 3.6 | Jan Min Temp | 8 | NR 03 | 5.7 |
| Curvature | 1.8 | NR08 + F04 | 5.5 | Redwood (%BA) | 4.8 |
| Subalpine | 1.1 | Curvature | 4.1 | Elevation | 4.1 |
| July Max Temp. | 0.9 | Insolation | 3.1 | January Precip. | 3.2 |
| Jan Precip. | 0.9 | July Precip | 1.5 | Oak Woodland | 2.6 |
| July Precip. | 0.8 | Jan Precip | 1.3 | July Max Temp | 1.3 |
| Insolation | 0.6 | Elevation | 0.4 | Insolation | 0.9 |
| Jan Min Temp | 0.3 | NR08 Core | 0.2 | Jan Min Temp | 0.7 |
| Northern Hdwd | 0.1 | Northern Hdwd | 0.2 | NR03 Core | 0.7 |
|  |  | Evergreen Hdwd | 0.1 | July precip. | 0.4 |
|  |  |  |  |  |  |

| **Western Cascades North** | | **Western Cascades Mid** | | **Western Cascades South** | |
| --- | --- | --- | --- | --- | --- |
| **Full Model** | **%** | **Full Model** | **%** | **Full Model** | **%** |
| NR05 Edge | 34.4 | NR09 Edge | 44.8 | NR 02 | 62.9 |
| NR 05 | 17.2 | NR09 + F01 | 13.9 | Slope Position | 17.8 |
| Slope Position | 13.0 | Curvature | 8.5 | Curvature | 4.7 |
| Curvature | 12.6 | Elevation | 7.6 | NR02 + F01 | 3.9 |
| Elevation | 8.0 | Northern Hdwd | 7.4 | Jan Min Temp | 3.9 |
| Jan Precip | 4.3 | Subalpine | 4.2 | Northern Hdwd | 1.9 |
| Northern Hdwd | 3.7 | Slope Position | 4.1 | Insolation | 1.5 |
| July Max Temp | 2.2 | Jan Min Temp | 2.4 | July Precip | 1.5 |
| Subalpine | 1.4 | NR 09 | 1.8 | January Precip | 0.9 |
| Insolation | 0.9 | July Precip | 1.5 | July Max Temp | 0.5 |
| July Precip | 0.9 | July Max Temp | 1.4 | Elevation | 0.5 |
| NR05 + F01 | 0.8 | Insolation | 1.0 |  |  |
| Jan Min Temp | 0.5 | NR09 Core | 0.7 |  |  |
| NR05 Core | 0.2 | Jan Precip | 0.7 |  |  |

| **Western Klamath** | | **Eastern Klamath** | | **Interior CA Coast Ranges** | |
| --- | --- | --- | --- | --- | --- |
| **Full Model** | **%** | **Full Model** | **%** | **Full Model** | **%** |
| Slope Position | 33.0 | NR01 | 28.3 | NR02 | 29.9 |
| NR01 Edge | 32.2 | Slope Position | 24.6 | NR02 Edge | 19.8 |
| NR01 | 10.9 | Douglas-fir | 12.1 | Slope Position | 12.4 |
| Curvature | 6.6 | Elevation | 9.2 | July Max Temp | 11.1 |
| January Precip | 6.1 | NR01 Edge | 6.8 | Curvature | 5.6 |
| July Precip | 4.4 | Insolation | 5.4 | NR02 + F04 | 4.9 |
| NR01 Core | 1.6 | Jan Precip | 4.9 | NR02 Core | 3.3 |
| Jan Min Temp | 1.3 | NR01 + F05 | 3.3 | July Precip | 2.6 |
| Elevation | 1.1 | Curvature | 2.2 | Jan. Precip | 2.4 |
| Insolation | 1.0 | July Max Temp | 1.2 | Insolation | 2.0 |
| July Max Temp | 0.8 | Jan Min Temp | 0.8 | Jan. Min Temp | 1.8 |
| NR01 + F03 | 0.5 | NR01 Core | 0.5 | Evergrn Hdwd | 1.7 |
| Oak Woodland | 0.2 | Oak Woodland | 0.2 | Pine | 1.3 |
| Evergrn Hrdwd | 0.2 | Pine | 0.2 | Oak Woodland | 0.7 |
|  |  | Subalpine | 0.1 | Elevation | 0.5 |

| **Eastern Cascades South** | | **Eastern Cascades North** | |
| --- | --- | --- | --- |
| **Full Model** | **%** | **Full Model** | **%** |
| NR07 + F03 | 18.4 | NR06 | 20 |
| NR 07 | 13.9 | Slope Position | 14.6 |
| NR07 Edge | 11.7 | Douglas-fir | 13.6 |
| Pine | 10.7 | Jan Min Temp | 10.6 |
| Douglas-fir | 10.7 | Elevation | 8.3 |
| Jan Min Temp | 9.5 | NR06 + F03 | 6.8 |
| Elevation | 5.4 | NR06 Edge | 5.7 |
| Slope Position | 4.6 | July Max Temp | 4.1 |
| NR07 Core | 4.5 | Subalpine | 4.0 |
| July Max Temp | 3.3 | January Precip | 3.3 |
| Insolation | 3.2 | Curvature | 2.9 |
| January Precip | 1.6 | Insolation | 2.7 |
| Curvature | 1.5 | July Precip | 2.1 |
| Subalpine | 0.6 | Pine | 1.5 |
| July Precip | 0.4 |  |  |

**Cross-validation of MaxEnt model**

Absolute values, of differences between the percentage of test and training site center locations classified within each of 10 RHS bins, were used for calculating means because without doing so, the positive and negative values within a modeling region will always have a mean of 0, and thus do not accurately represent overall differences between full and cross-validated models. There was an inverse (negative logarithmic) relationship between sample size of spotted owl sites and mean difference in absolute value (r^2^ = 0.537, P = 0.01). Nonetheless, the magnitude of differences was generally quite low. For example, 39% of the 110 differences were <2.0, 81% of the differences were <5.0, and only 7% of the differences were >7.0 (absolute value in each case; Table H).

**Table H.** **Results from cross-validation tests, showing absolute values of differences (% classified by full model - % classified in cross-validated model) in percentage of owl locations classified into each of 10 relative habitat suitability (RHS) bins among modeling regions. Cross-validation was done by randomly removing 25% of the presence locations, training the model with the remaining 75% of locations, then classifying the withheld 25% (test locations) each of ten times. Values below represent mean absolute differences between full and cross-validated models.**

|  | **Absolute value of differences** | | | | | | | | | | |
| --- | --- | --- | --- | --- | --- | --- | --- | --- | --- | --- | --- |
| **RHS Bin** | **ECN** | **ECS** | **ICC** | **KLE** | **KLW** | **NCO** | **ORC** | **RDC** | **WCC** | **WCN** | **WCS** |
| 0-9.999 | 5.2 | 4.8 | 3.9 | 3.0 | 0.9 | 5.2 | 3.3 | 1.9 | 7.9 | 11.1 | 1.7 |
| 10-19.999 | 4.4 | 4.6 | 6.1 | 1.1 | 5.0 | 0.2 | 3.3 | 3.1 | 1.9 | 4.2 | 1.7 |
| 20-29.999 | 3.3 | 1.0 | 3.1 | 4.6 | 1.4 | 1.1 | 0.2 | 1.4 | 4.0 | 3.4 | 2.6 |
| 30-39.999 | 2.8 | 4.5 | 0.9 | 3.7 | 2.8 | 0.5 | 3.0 | 3.5 | 0.9 | 1.3 | 2.6 |
| 40-49.999 | 2.8 | 7.9 | 2.5 | 2.4 | 0.0 | 4.5 | 0.7 | 5.2 | 3.7 | 1.3 | 0.8 |
| 50-59.999 | 3.1 | 1.0 | 3.6 | 4.4 | 0.8 | 0.1 | 6.2 | 6.1 | 4.4 | 4.5 | 5.5 |
| 60-69.999 | 5.2 | 3.1 | 7.0 | 7.3 | 0.3 | 1.4 | 1.9 | 3.3 | 9.9 | 5.3 | 8.1 |
| 70-79.999 | 3.5 | 9.7 | 3.4 | 0.6 | 4.0 | 10.2 | 3.4 | 6.8 | 1.7 | 5.8 | 2.9 |
| 80-89.999 | 1.5 | 2.5 | 2.1 | 1.0 | 1.1 | 0.2 | 2.0 | 2.2 | 4.0 | 6.8 | 1.2 |
| 90-100 | 0.3 | 2.4 | 0.4 | 0.3 | 0.1 | 0.8 | 0.4 | 0.5 | 1.0 | 1.1 | 0.1 |
| **Mean** | **3.2** | **4.1** | **3.3** | **2.8** | **1.6** | **2.4** | **2.4** | **3.4** | **3.9** | **4.5** | **2.7** |

The rank correlation between bin rank and cross-validated data SOS for each of the 11 modeling regions averaged 0.989 (range 0.963-0.996). Differences between AUC values from the full model and AUC from the cross-validated models’ test data averaged 0.0214 (range 0.0088 – 0.04) for the 11 modeling regions.

**Table I. Distribution of relative habitat suitability values (RHS) within each modeling region and range-wide. Values represent the percentage of the modeling region comprised of each RHS bin.**

| **RHS** | **ECN** | **ECS** | **ICC** | **KLE** | **KLW** | **NCO** | **ORC** | **RDC** | **WCC** | **WCN** | **WCS** | **Range-wide** |
| --- | --- | --- | --- | --- | --- | --- | --- | --- | --- | --- | --- | --- |
| 0-9.999 | 56.31 | 59.42 | 40.11 | 41.13 | 22.18 | 70.88 | 55.50 | 30.41 | 62.96 | 77.22 | 38.68 | 52.19 |
| 10-19.999 | 14.91 | 14.24 | 12.58 | 13.49 | 14.81 | 12.07 | 12.72 | 11.95 | 9.33 | 7.16 | 8.27 | 12.02 |
| 20-29.999 | 9.54 | 8.71 | 12.77 | 11.67 | 15.47 | 5.88 | 9.34 | 12.62 | 6.25 | 4.37 | 11.27 | 9.50 |
| 30-39.999 | 6.68 | 5.92 | 11.60 | 10.47 | 14.66 | 3.74 | 7.53 | 13.27 | 5.67 | 3.43 | 12.11 | 8.22 |
| 40-49.999 | 4.82 | 4.24 | 9.56 | 9.16 | 13.61 | 2.62 | 5.92 | 13.09 | 5.51 | 2.79 | 11.97 | 7.13 |
| 50-59.999 | 3.43 | 3.14 | 6.85 | 7.25 | 11.42 | 2.00 | 4.59 | 10.62 | 5.07 | 2.20 | 10.62 | 5.74 |
| 60-69.999 | 2.27 | 2.28 | 4.25 | 4.72 | 6.12 | 1.60 | 2.88 | 6.07 | 3.71 | 1.64 | 6.33 | 3.61 |
| 70-79.999 | 1.37 | 1.50 | 1.94 | 1.89 | 1.64 | 0.98 | 1.24 | 1.82 | 1.37 | 0.94 | 0.73 | 1.33 |
| 80-89.999 | 0.63 | 0.51 | 0.34 | 0.22 | 0.08 | 0.24 | 0.27 | 0.15 | 0.12 | 0.26 | 0.00 | 0.25 |
| 90-100 | 0.06 | 0.03 | 0.00 | 0.00 | 0.00 | 0.00 | 0.01 | 0.00 | 0.01 | 0.00 | 0.00 | 0.01 |

**Table J. Size (millions of ha) and percentage of total area within relative habitat suitability (RHS) bins among candidate critical habitat networks for the northern spotted owl. For example, 21.5% of the total area of the owl’s geographic range that exists within 0-9.99 RHS bin and 58.2% of the total area of the owl’s geographic range that exists within the ≥90 RHS bin occur within the NWFP. Similarly, 17.7% of the total area of the owl’s geographic range that exists within 0-9.99 RHS bin and 80.6% of the total area of the owl’s geographic range that exists within the ≥90 RHS bin occur within Comp 11. 2008 CH = 2008 Critical Habitat; NWFP = Northwest Forest Plan, and Comp = Composite; Z = Zonation-derived networks. The number after Z represents the percentage of habitat value and ALL = no prioritization of lands included, whereas PUB = public lands were prioritized and non-public lands were only included if the goal could not be met with public lands.**

| **Relative Habitat Suitability Bin** | | | | | | | | | | | |
| --- | --- | --- | --- | --- | --- | --- | --- | --- | --- | --- | --- |
| **Network** | **Size** | **0-9.99** | **10-19.99** | **20-29.99** | **30-39.99** | **40-49.99** | **50-59.99** | **60-69.99** | **70-79.99** | **80-89.99** | **≥90** |
| 2008 CH | 5.82 | 17.7% | 22.5% | 27.7% | 32.9% | 37.8% | 42.9% | 48.7% | 56.3% | 63.4% | 54.3% |
| NWFP | 6.63 | 21.5% | 26.3% | 31.4% | 36.1% | 40.7% | 45.9% | 51.4% | 57.1% | 63.5% | 58.2% |
| Comp 1 | 9.30 | 22.0% | 41.3% | 50.8% | 59.7% | 68.8% | 78.6% | 86.3% | 91.1% | 96.3% | 97.8% |
| Comp 2 | 7.02 | 20.6% | 27.1% | 31.2% | 36.9% | 44.9% | 57.0% | 70.1% | 80.5% | 90.1% | 94.8% |
| Comp 3 | 9.04 | 25.0% | 39.6% | 45.9% | 53.2% | 61.3% | 70.6% | 77.7% | 81.3% | 80.8% | 72.6% |
| Comp 4 | 8.79 | 22.7% | 37.2% | 44.9% | 53.4% | 62.9% | 73.3% | 80.9% | 85.4% | 90.7% | 85.5% |
| Comp 5 | 8.22 | 22.1% | 34.2% | 41.0% | 48.7% | 57.5% | 67.5% | 75.3% | 79.8% | 82.9% | 76.0% |
| Comp 6 | 8.09 | 18.1% | 31.4% | 42.9% | 53.4% | 64.1% | 75.0% | 82.8% | 86.7% | 90.3% | 81.4% |
| Comp 7 | 7.73 | 17.8% | 29.4% | 39.5% | 49.4% | 60.1% | 71.9% | 81.3% | 86.6% | 90.4% | 81.4% |
| Comp 8 | 7.02 | 17.5% | 26.2% | 34.7% | 43.1% | 52.4% | 62.9% | 71.6% | 77.7% | 80.9% | 73.9% |
| Comp 9 | 7.55 | 17.8% | 28.3% | 38.0% | 47.7% | 58.3% | 70.1% | 79.9% | 85.5% | 89.1% | 80.4% |
| Comp 10 | 7.64 | 17.8% | 28.2% | 38.2% | 48.5% | 59.9% | 72.0% | 81.2% | 86.0% | 89.5% | 80.9% |
| Comp 11 | 7.52 | 17.7% | 27.8% | 37.5% | 47.5% | 58.5% | 70.4% | 79.6% | 84.5% | 88.6% | 80.6% |
| Z30ALL | 5.61 | 0.6% | 4.6% | 10.7% | 19.6% | 33.3% | 53.3% | 73.9% | 88.7% | 95.4% | 100.0% |
| Z30PUB | 5.57 | 0.9% | 6.0% | 12.5% | 21.3% | 33.7% | 51.4% | 69.7% | 83.0% | 89.7% | 90.7% |
| Z50ALL | 7.80 | 2.4% | 15.1% | 29.7% | 45.8% | 63.4% | 80.7% | 91.9% | 97.5% | 99.3% | 100.0% |
| Z50PUB | 7.82 | 3.9% | 20.7% | 35.0% | 47.3% | 60.9% | 75.4% | 85.3% | 90.1% | 92.2% | 91.0% |
| Z70ALL | 10.55 | 8.2% | 38.2% | 59.1% | 74.7% | 87.2% | 95.4% | 98.7% | 99.8% | 100.0% | 100.0% |
| Z70PUB | 11.24 | 24.6% | 47.4% | 58.5% | 70.2% | 81.2% | 89.8% | 94.5% | 96.7% | 98.3% | 99.7% |

**HexSim calibration**

HexSim estimates of total population size were averaged among the five replicates. In comparison, the total estimated female spotted owls in those DSAs based on the largest number recorded during field surveys between 1996 and 2006 was 778. The average of the three largest population years (using data from 1996-2006) for total estimated spotted owl females was 756. The mean of the highest three years (1996-2006) was used for comparison in order to reduce the chance that a single year was uncharacteristic of the DSA. For all DSA populations combined, HexSim’s mean total population size was 89% of the empirical population estimate. Differences in the number of female spotted owls on the eight DSAs estimated from field surveys and those estimated from HexSim ranged from 5% to 47%, with a mean absolute percentage difference of 26% (S1 Fig). Subsequent modifications of the spotted owl HexSim model did not eliminate these differences.

The distributions of natal dispersal distances for 328 banded female spotted owls [102] were similar to 850,000 natal dispersal events recorded during a 250-time step HexSim simulation (S2 Fig). The majority of both observed and simulated dispersal distances were between one and 25 km, however, compared to [102], about 10% fewer simulated dispersal distances were greater than 10 km and 20% fewer were greater than 25 km.

**Sensitivity analyses**

Varying survival rates by 2.5% had the most dramatic impacts on population sizes. A reduction in survival of 2.5% had a much larger negative effect on population size (a 250-fold decrease) than did a 2.5% increase (a 2.24-fold increase in population size) (Table K). Ten percent increases and decreases in reproduction also resulted in relatively large effects on population sizes; again with reductions of 10% showing larger negative effects (8.6 fold decrease) than 10% increases showed (a 1.73 fold increase). In large part, the sensitivity analysis evaluations at the modeling region scale showed very similar patterns to those at the geographic range scale (Table K).

**Table K. Sensitivity analyses of the northern spotted owl HexSim model based on modifying individual parameters (see Table F for specific modifications of each parameter). Values presented are ratios of population sizes between parameter-value modified conditions and the base NSO HexSim model at time-step 350 for the entire geographic range and within each of the eleven modeling regions. Ratios near 1.0 suggest little or no difference. Mean MR = mean of the ratios for all modeling regions, with the associated standard errors.**

| **Area** | **Metric** | **1A** | **1C** | **2A** | **2C** | **3A** | **3B** | **3C** | **3D** | **4A** | **4C** | **4D** | **5A** | **5C** | **6A** | **6C** | **7A** | **7C** | **8A** | **8C** | **9A** | **9C** |
| --- | --- | --- | --- | --- | --- | --- | --- | --- | --- | --- | --- | --- | --- | --- | --- | --- | --- | --- | --- | --- | --- | --- |
| Range | N_350_ | 1.005 | 0.976 | 1.004 | 1.001 | 1.216 | 1.620 | 0.836 | 0.640 | 1.612 | 0.084 | 0.772 | 1.014 | 0.999 | 0.004 | 2.242 | 0.116 | 1.727 | 1.049 | 0.957 | 0.992 | 0.992 |
| ECN | N_350_ | 0.893 | 0.946 | 0.947 | 1.033 | 1.187 | 1.759 | 0.802 | 0.544 | 1.722 | 0.764 | 0.682 | 0.946 | 0.919 | 0.003 | 4.374 | 0.053 | 2.904 | 1.074 | 0.832 | 0.924 | 0.979 |
| ECS | N_350_ | 0.992 | 0.961 | 1.010 | 1.011 | 1.216 | 1.532 | 0.854 | 0.679 | 1.560 | 0.846 | 0.744 | 1.016 | 1.015 | 0.009 | 1.838 | 0.198 | 1.503 | 1.014 | 0.977 | 0.956 | 1.011 |
| ICC | N_350_ | 1.003 | 0.973 | 0.996 | 0.984 | 1.189 | 1.569 | 0.843 | 0.650 | 1.563 | 0.806 | 0.801 | 1.016 | 1.001 | 0.002 | 1.738 | 0.163 | 1.364 | 1.046 | 0.969 | 0.981 | 0.991 |
| KLE | N_350_ | 1.005 | 0.990 | 1.002 | 1.000 | 1.201 | 1.552 | 0.843 | 0.663 | 1.536 | 0.811 | 0.785 | 1.038 | 0.973 | 0.002 | 1.823 | 0.107 | 1.459 | 1.039 | 0.964 | 0.979 | 0.996 |
| KLW | N_350_ | 1.005 | 0.975 | 1.004 | 0.993 | 1.193 | 1.560 | 0.837 | 0.652 | 1.555 | 0.807 | 0.791 | 1.008 | 0.990 | 0.002 | 1.699 | 0.108 | 1.385 | 1.088 | 0.956 | 0.997 | 0.983 |
| NCO | N_350_ | 1.025 | 0.891 | 0.978 | 1.023 | 1.207 | 1.886 | 0.749 | 0.609 | 1.872 | 0.741 | 0.646 | 0.896 | 1.198 | 0.001 | 4.753 | 0.036 | 3.305 | 1.085 | 0.909 | 0.925 | 1.044 |
| ORC | N_350_ | 1.014 | 0.892 | 1.040 | 1.025 | 1.163 | 1.483 | 0.851 | 0.654 | 1.570 | 0.856 | 0.805 | 0.995 | 1.046 | 0.001 | 3.063 | 0.034 | 2.376 | 0.841 | 0.967 | 1.001 | 0.967 |
| RDC | N_350_ | 1.006 | 0.979 | 0.999 | 0.996 | 1.274 | 1.772 | 0.811 | 0.589 | 1.750 | 0.762 | 0.746 | 1.011 | 0.990 | 0.003 | 2.009 | 0.182 | 1.491 | 0.975 | 0.909 | 0.979 | 1.012 |
| WCC | N_350_ | 0.930 | 0.936 | 0.991 | 0.987 | 1.329 | 1.916 | 0.914 | 0.447 | 2.181 | 0.692 | 0.627 | 1.033 | 0.857 | 0.000 | 5.415 | 0.053 | 3.512 | 1.086 | 0.979 | 0.865 | 0.944 |
| WCN | N_350_ | 0.801 | 0.692 | 0.749 | 0.946 | 0.995 | 2.473 | 0.585 | 0.263 | 2.092 | 0.503 | 0.584 | 1.038 | 0.776 | 0.002 | 9.259 | 0.013 | 4.308 | 0.664 | 0.801 | 0.677 | 1.015 |
| WCS | N_350_ | 1.030 | 1.024 | 1.019 | 1.027 | 1.282 | 1.738 | 0.843 | 0.643 | 1.679 | 0.816 | 0.741 | 1.026 | 1.026 | 0.000 | 3.526 | 0.043 | 2.695 | 1.182 | 1.000 | 1.063 | 0.985 |
|  | Mean MR | 0.973 | 0.933 | 0.976 | 1.002 | 1.203 | 1.749 | 0.812 | 0.581 | 1.735 | 0.764 | 0.723 | 1.002 | 0.981 | 0.002 | 3.591 | 0.090 | 2.391 | 1.009 | 0.933 | 0.941 | 0.994 |
|  | SE | 0.021 | 0.027 | 0.024 | 0.008 | 0.026 | 0.085 | 0.026 | 0.038 | 0.068 | 0.03 | 0.023 | 0.013 | 0.033 | 0.0007 | 0.697 | 0.02 | 0.311 | 0.043 | 0.019 | 0.03 | 0.008 |
