# Supplementary figures and images for: Conservation planning for species recovery under the Endangered Species Act: A case study with the Northern Spotted Owl
Source: PLoS One. 2019 Jan 14;14(1):e0210643. doi: 10.1371/journal.pone.0210643 (PMC6331132; doi:10.1371/journal.pone.0210643)

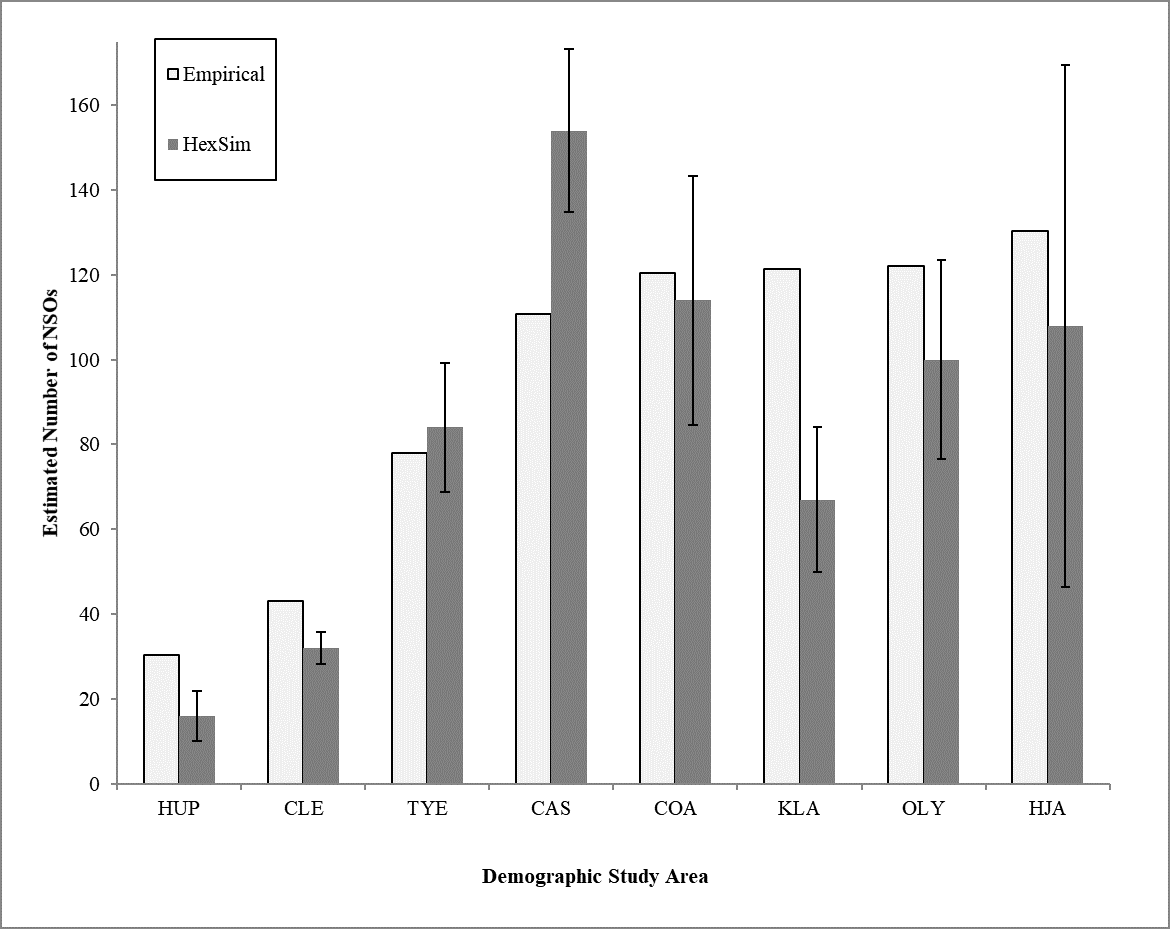

Supplement: S1 Fig — (TIF) [file pone.0210643.s002.tif]

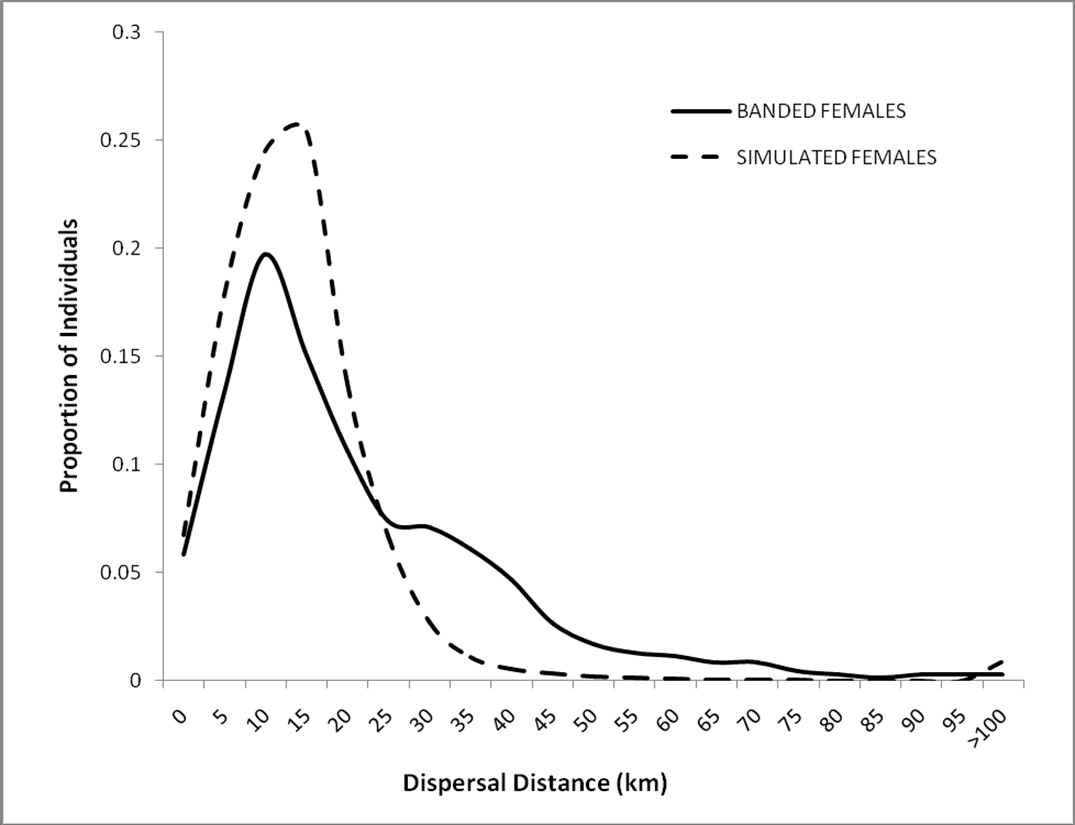

Supplement: S2 Fig — (TIF) [file pone.0210643.s003.tif]
